# “Measuring the health and fiscal outcomes of solid waste management operations by intergovernmental arrangements: The case of public consortia in Brazil”

**DOI:** 10.1016/j.heliyon.2024.e26032

**Published:** 2024-02-18

**Authors:** Yan Nonato Cattani, Raquel Pereira Pontes, Diego Camargo Botassio, Daniel Kiyoyudi Komesu, Rodolfo Gomes Benevenuto, Mario Henrique Ogasavara

**Affiliations:** a PSP HUB and ESPM, Brazil; b PSP HUB and Unisinos, Brazil; c PSP HUB, Brazil; d ESPM, Brazil

**Keywords:** Solid waste management, Analytical methodologies, Public consortia, Staggered difference-in-differences, Cooperative intergovernmental arrangements, Impact evaluation

## Abstract

This study estimates the health-related and public expenditure impacts of the solid waste services provided by public consortia in Brazilian Municipalities from the enactment of Public Consortia law (2005) to 2019. To conduct the analysis, we applied the econometric method of staggered difference-in-differences to publicly available datasets at the municipality level. The results show that the operation of solid waste services by public consortia had statistically significant effects in reducing hospitalizations caused by Schistosomiasis, Diarrhea/gastroenteritis (up to 5 years age) and other intestinal diseases. The results also indicate a positive impact on the reduction of environmental expenses in treated municipalities, supporting the idea that a Solid Waste Consortium can serve as a local coordinator and improve health and fiscal indicators simultaneously. The findings provide quantitative evidence that policymakers at the local and regional level can use to better understand the benefits of adhering to public consortia when preparing new investments and operation developments for this sector. This paper contributes to the literature of applied research in solid waste by shedding light on the underexplored theme of the intergovernmental cooperative arrangements, which can be instrumental in accelerating and enhancing the development of solid waste services.

## 1 Introduction

In 2022, Brazil generated approximately 81.8 million tons of urban solid waste, equivalent to 224,000 daily tons or 1.043 kg per inhabitant [1]. With the world's sixth-largest population, the sheer volume of solid waste generated in Brazil is a major concern. Of this total, 39.5% (30.2 million tons) are still inadequately disposed in controlled dumps and landfills, as reported by the Brazilian Association of Public Cleaning and Special Waste Companies [1]. Population growth intensified human activities and improved living standards have led to an exponential increase in the amount of solid waste generation and the change in its characteristics, posing a significant problem for public administrations. Compounding this issue, inadequate management of solid waste from generation to final disposal (e.g., in open dumps or even in watercourses) results in environmental, social, economic, and public health risks [2,3].

Sanitation and solid waste services are constitutionally guaranteed rights under the 1988 Constitution of the Federative Republic of Brazil (CRFB). As determined in art. 30 of the CRFB, the responsibility for this type of public service is assigned to the local level, namely the municipalities [4,5]. Nevertheless, the provision of solid waste management (SWM) services can be executed either directly by the public administration or under a concession or permission regime.

In 2007, Law No. 11,445/2007 [47] established national guidelines for the sanitation sector, aiming at providing and universalizing public sanitation services in the country, including SWM practices [5]. In 2010, the National Solid Waste Policy (“Política Nacional de Resíduos Sólidos,” PNRS) [49] mandated treatment before final disposal, with a priority order of actions encompassing non-generation, reduction, reuse, recycling, composting, recovery, and energy use of solid waste. In this regard, further regulatory instructions were also stablished by Decree No. 10,936/2022, which established the National Solid Waste Plan (“Plano Nacional de Resíduos Sólidos,” PLANARES) [68].

As depicted by UNDP & ME [4] and Cattani et al. [6], the provision of urban SWM services by cooperative intergovernmental arrangements (CIA) [54] can benefit public spending and improve health-related indicators through a most robust type of CIA: the public consortia (PC), created by the enactment of Law 11.107/2005 [46] and its regulatory decree 6.017/2007 [67].

Therefore, this paper aims to contribute to the literature on applied research in SWM by specifically analyzing the measurable outcomes of SWM services provision by PC. This study also contributes to the SWM body of knowledge by bringing a rigorous econometric model predominantly seen only in other areas such as education, health, poverty, and wealth distribution research [[7], [8], [9], [10]].

Following this introduction, the structure of this paper includes a literature review on the typical outcomes of SWM, a methodological part describing the data and the econometric model applied, and finally, the findings derived from this research, along with discussions and policy recommendations.

## 2 Literature review

This literature review is conducted through two main directives. The first stems from the findings of Cattani and Ogasavara [11] and UNDP & ME (2022a), encompassing a comprehensive review of the approach of public consortia in Brazil and the assessment of public services they deliver. The second directive, based on the technical evaluation of the investment needs in the solid waste sector in Brazil, conducted by the Ministry of Economy of Brazil [5], is included as part of the literature review. Therefore, this section aims to outline key studies that connect additional factors with investment information and techniques regarding waste management, with a primary focus on sanitary aspects.

The adequate disposal of solid waste impacts a wide range of outcomes in public policies, influencing economic effects, productivity, education, and health indicators. Daly and Farley [12] assert that environmental contamination and the accumulation of high-entropy materials constrain economic growth. Climate change is also a significant concern, attributed to the emission of polluting gases (Sanjeevi & Shahabudeen, 2015). According to the Greenhouse Gas Emissions Estimation System [13], Brazil ranked as the seventh-largest global emitter of greenhouse gases, with 2.17 billion gross tons of carbon dioxide equivalent (tCO2e) in 2019, contributing to 3.4% of global emissions.

Saiani, Mendonça, and Kuwahara [14] bring up the debate on human development, referencing Sen [15,16], Nussbaum [17], and Robeyns (2005). The authors highlight how the lack of proper waste management can act as a constraint on individual liberties, impacting the capacity to make life choices. This perspective can influence various outcomes, such as children's academic performance and its repercussions on labor market productivity [9,[18], [19], [20]].

In terms of health indicators, the literature examines the impact on morbidity and public health expenditures. Brundtland [21] notes that infectious diseases contribute significantly to the death toll, with only six responsible for over 90% of total global diseases. Malaria stands out as the most fatal parasitic disease, while other relevant protozoal parasites include trypanosomiasis, leishmaniasis, amoeba, hookworm, ascaris, schistosomiasis, onchocerciasis, among others [22].

**Fig. 1:**
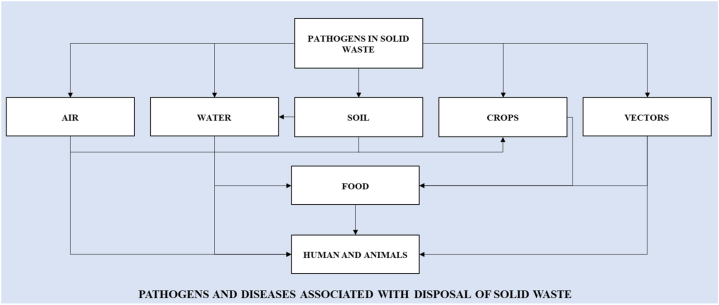
Transmission channels of pathogens.

Source: Epstein [22].

Some authors highlight that children under five years old constitute a significant source of contamination due to ingestion of soil or contaminated materials ([22]; Saiani, Mendonça & Kuwahara, 2020). The transmission channels of pathogens are illustrated by Epstein [22] in Fig. 1.

As emphasized by Epstein [22], the primary source of pathogens lies in soil and water environments, as soil can retain chemical, physical, and biological characteristics. Children are particularly vulnerable to the mentioned vectors, especially concerning viral infections (e.g. yellow fever, dengue fever, and encephalitis), bacterial diseases (e.g. Rickettsia typhi and Yersinia species), and parasitic infections (e.g. helminths or protozoa) [22].

Saiani [23] analyzed the determinants and effects of privatizing essential sanitation services, specifically focusing on Brazil's water supply and sewage collection. In terms of impacts on epidemiological indices, i.e., morbidity and mortality, the results indicate that the decentralized private provision model decreases the incidence of these indicators. Contrary to the hypothesis of a *cost-quality trade-off* in the private provision of public services, particularly sanitation, these findings suggest that political competition influences mayors' electoral risk, and privatization positively impacts access and service quality, thereby reducing morbidity and mortality indicators.

Another paper by Saiani, Mendonça, and Kuwahara [14] argues that health literature indicates a positive relationship between the coverage and quality of environmental sanitation services. The authors conducted econometric data estimations in Brazilian and São Paulo municipalities. The study supported the hypotheses that the existence and quality of sanitary landfills, along with the exportation of solid waste, lead to better health indicators. They identified an average reduction of 1.25% in hospital morbidity associated with the existence of sanitary landfills and 0.75% due to their quality. Robust effects were also observed in children, the elderly, and waterborne diseases, which are more directly and immediately affected by service conditions. Finally, the authors identified common diseases and organized Table 1 with the following content.

**Table 1:** Forms of Environmental Exposure to risk factors.

Forms of Environmental Exposure to risk factors	Categories	Diseases
**Contact with Waste or Contaminated Soil**	Feco-Oral Transmission	Diarrhea; Gastroenteritis; Typhoid Fever; Paratyphoid fever; Bacillary dysinteria; Cholera; Jersindiasis; Hepatitis A; Amibic desinteria; Ascariasis; Tricuriasis
**Contact with vector**	Skin Penetration	Tetanus; Hookworm; Scabies; Serpiginous dermatitis; Piodermites (impetigo, folliculitis, boil, erysipelas, cellulitis, bacterial conjunctivitis)
	Mechanical Vector Transmission	Typhoid Fever; Cholera; Giardiasis; Bubonic plague; Diarrhea
	Transmission by Biological Vector	Leishmaniasis; Black vomit; Malaria; Dengue fever; Filariasis; Cysticercosis; Toxoplasmosis; Teniasis; Diarrhea
**Contact with Contaminated Water**	Skin Penetration	Schistosomiasis
	Ingestion	Helminth Infections, Teniasis, and Cysticercosis
	Chemical Contamination	Delayed growth of children (lower weight and height); Bladder, Lung, Esophagus, Stomach, Large, Reuse, and Breast Cancer; Low Birth Weight

## 3 Data and methods

This section will present the employed econometric model, providing details on the specific Staggered Difference-in-Difference methodology, particularly the one developed by Calaway & Santana (2021). Additionally, a description of all information compiled for the regressions and its sources will be provided.

### 3.1 Econometric model

The Difference-in-Differences (DID) method measures the causal impact of an intervention by comparing outcomes between a treatment group and a control group over time. This approach is widely adopted in economics, public policy, and health research. It helps mitigate selection biases (where the groups differ in ways that affect the outcome) and endogeneity (where external factors influence both the intervention and the outcome), thereby enhancing the credibility of causal inferences. A key strength of DID is its ability to utilize global, routinely updated datasets like censuses for pre- and post-intervention analysis. Typically, DID involves a framework with two groups and two time periods to assess the effects of interventions, as noted by Ref. [26].

In assessing infrastructure impacts, the Staggered Difference-in-Differences (Staggered DID) method is gaining popularity in academic and policy circles, noted for its wide applicability and flexible hypotheses [27]. It surpasses traditional DID by accommodating staggered treatment times and addressing anticipation or spillover effects. It provides a more refined approach to analyzing complex policy effects, thus offering a deeper understanding of the impacts of staggered interventions.

Callaway and Sant'Anna (2021) delve into the use of binary treatment methodologies in their research. They specifically focus on scenarios where municipalities, once included in the treatment group, remain there for the entire study duration. To form the control group, they introduce two distinct categories: one consisting of municipalities *not yet treated* but might be in the future, and the other comprising municipalities that are never expected to be treated (*never treated*). This approach is particularly crucial in studies with smaller sample sizes, where the dynamics of treatment can significantly impact the results. Notably, their method facilitates conditional adherence to the parallel trend assumption - a key factor in causal inference - based on the covariates included in the model. This enhances both the robustness and applicability of their approach.^1^

As various Brazilian municipalities have implemented solid waste policies with diverse complexities of technological routes and legal/illegal dumping sites at different times through public consortia, we can feasibly estimate the Staggered DID model as proposed by Callaway and Sant'Anna (2021). In applying the DID models, we followed the frameworks primarily outlined by Nascimento et al. [28], which are detailed in Table 2.

**Table 2:** Representation of DID models: dependent and independent variables.

	References	Variable
**Dependent Variables: Outcomes**	Nascimento et al. [28], Cairncross and Feachem [29], Mara and Feachem [24], Saiani [23], Saiani, Mendonça and Kuwahara [14], Azevedo et al. [30], Mendonça [25]	Incidence of non-standard fecal coliforms
		Outpatient Production - Primary Care
		Outpatient Production - Medium Complexity
		Outpatient Production - High Complexity
		Schistosomiasis
		Hepatitis
		Leptospirosis
		Dengue
		Acute Chagas disease
		Malaria
		Dermatosis
		Yellow fever
		Hantavirus
		Leishmaniasis
		Whooping
		Diarrhea and infectious gastroenteritis
		Other intestinal diseases
		Delayed Fetal Growth and Fetal Malnutrition
	Silvestre [31], UNDP & ME [[41], [65], [66]]	Sanitation Expenditures
		Environmental Expenditures
**Independent Variables: covariates**	Nascimento et al. [28]	Population
		Population/Km^2^
		GDP
		GDP per Capita
		Unemployment
		Access to water
		Other Public Consortia
	Bel & Warner [32], Nascimento et al. [28]	Health expenditures
		Municipality expenses
		Municipality revenue
		Other Revenues
	Nascimento et al. [28], Saiani [23]	PPP in Sanitation
		Mayor Re-election

From our choice of the Staggered DID method, as proposed by Callaway and Sant'Anna (2021), we now define the model specification that will be estimated. This decision is based on the literature review. The following paragraphs will detail the model's setup and the rationale behind its selection. The model specification is (Equation (1)):

(1) $$Y_{it}={SWC}_{i}\times \sum_{t}\beta I\left(t\geq \tau \right)+\alpha X_{it}+\theta_{i}+\theta_{t}+\varepsilon_{it}$$

whereas: $Y_{ivt}$ is *outcome* of interest of municipality i at year t; ${SWC}_{i}$ is a *binary variable*, which will be equal to 1 if municipality i is member of a solid waste CIA (SWC) (with full or partial services^2^) at some point, and equal to 0 otherwise; *I*(*t* ≥τ) is an indicator function that is equal to 1 for the entire period after the municipality i is treated for the first time (period τ); β is the effect of interest and corresponds to the SWC effect; $X_{it}$ is the covariate vector; $\theta_{i}$ and $\theta_{t}$ are the fixed effects of municipality and year, respectively; $\varepsilon_{it}$ is idiosyncratic error.

To estimate the pre-treatment and post-treatment effects, the following equation (Equation (2)) was developed:

(2) $$Y_{it}={SWC}_{i}\times \sum_{\tau \neq -1}\beta_{\tau }I\left(t-\text{first}\;\text{treat}=\tau \right)+\alpha X_{it}+\theta_{i}+\theta_{t}+\varepsilon_{it}$$

In our model, the following terms are used: I(t−firsttreat=τ) is an indicator function that mediates the time relative to the year of implementation of the SWC in municipality i; $\beta_{\tau }$ is the effect of interest. The coefficient for the year prior to the SWC implementation in municipality *i* is omitted (i.e. $\beta_{\tau =-1}$ is not included); the other terms were described earlier.

To estimate the effects of SWC, we took into account the potential *spillovers* between the municipalities that received the SWC at different points in time. To address this, we clustered the errors using both municipality and SWC clusters. We defined “treated” the Brazilian municipalities that received SWC at points in time, and as “control” (never treated), we considered Brazilian municipalities that did not have an SWC during the analyzed period.

### 3.2 Database

In contrast to Jones et al. [33], who selected vectors based on evidence of disease transmission, we chose to utilize available data from official databases to conduct the ex-post analysis.

Five sources of information were utilized to collect data related to the dependent variables analyzed in this study. The first is the Brazilian National Sanitation Information System (Sistema Nacional de Informações de Saneamento - SNIS)[63]^2^, where water quality indicators and the incidence of non-standard fecal coliform analyses were scrutinized. This analysis considered the hypothesis that proper waste management disposal influences water quality.

The second source is the Brazilian Unified Health System database (DATASUS), which provides hospital information. This includes four types of outpatient production: (i) total approved procedures of outpatient production; (ii) primary care approved procedures of outpatient production; (iii) medium complexity approved procedures of outpatient production, and; (iv) high complexity approved procedures of outpatient production.

The third source is the Brazilian Notifiable Diseases Information System (SINAN), which provides notifications of diseases that do not necessitate treatment at the health care unit (i.e. cases that can be treated at the patient's home).

The fourth source is the Brazilian Unified Health System database (DATASUS) related to epidemiology and morbidity. The analysis included “Hospitalizations by Place of Residence” due to diarrhea and infectious gastroenteritis, other intestinal diseases, as well as delayed fetal growth and malnutrition [[56], [57], [58]].

The fifth source is the Brazilian National Treasury (Secretaria do Tesouro Nacional, STN). The amount of expenditure for each municipality was also analyzed to ascertain whether SWC has a downsizing effect on sanitation and environmental budgetary expenses, potentially indicating economic scale gains.

To control for potential confounding factors and enhance the accuracy of estimated relationships, the model incorporates several pertinent covariates, as outlined in Table 3.

**Table 3:** Covariates applied in the model.

Database	Variable	Description
MDR - Regional Development Ministry: SNIS [63]	Access to water	Coverage in access to water (% total population)
IBGE – Brazilian Institute of Geography and Statistics: IBGE Cidades [69]	Population	Local inhabitants
	Population/Km^2^	Local inhabitants are divided by area.
	GDP	Local Gross Domestic Product
	GDP per Capita	Local Gross Domestic Product divided by inhabitants
MTE – Labor Ministry: RAIS-CAGED (Formal workforce inquiries system) [61]	Unemployment	1- (Formal workforce/Population)
CNM and MMA – National Municipality Confederation and Environment Ministry [55][60]	Other Public Consortia	Participation in other Public Consortia, excluding Solid Waste and Sanitation sectors
ABCON-SINDICON – National Association and Union of Private Concessionaires of Public Water and Sewage Services: Annual Reports [40]	PPP in Sanitation	Public Private participation in the sanitation sector
TSE – Superior Electoral Court [70]	Mayor Re-election	Verifies if there was at least one mayor re-election during the period
STN (MF): National Treasure – Economy Ministry [64]	Health expenditures	Health expenditures divided by population
	Municipality expenses	Current Expenses divided by Current Revenues
	Other Revenues	Intergovernmental Transfers divided by Current Revenues
	Municipality revenues	Tax revenues divided by current revenues

**Fig. 2:**
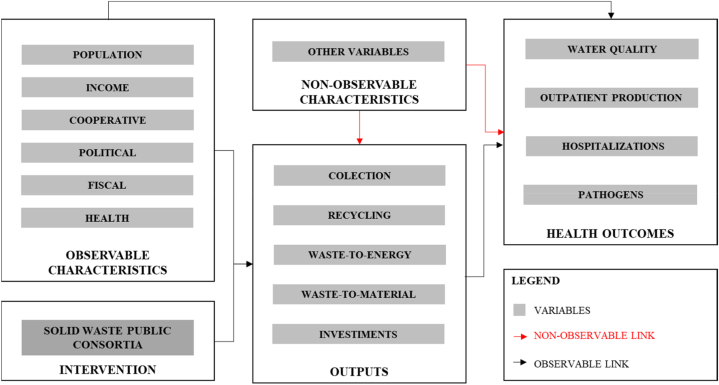
Directed Acyclic graph of the proposed model.

Source: elaborated by authors.

Finally, to summarize the hypothesis regarding the causal channels among the formation of public consortia in the provision of solid waste services, the pre-existing characteristics, outputs, and the variables of interest (outcomes), a Directed Acyclic Graph (DAG) is presented in Fig. 2.

The DAG illustrates how solid waste services can impact the population's health ([23,24,28,29,[59]]; Saiani, Mendonça & Kuwahara, 2015; [25,30]) and expenditures [34]. Subsequently, these outputs influence the variables of interest: outpatient production, water quality indicators, hospitalization, and notifiable diseases. However, pre-existing observable characteristics of citizens, such as the quality and quantity of the solid waste sector, and health indicators related to the lack of basic SWM, GDP, and population size, also impact the dynamics of the variables of interest.

## 4 Results and discussions

The results of the parallel trends analysis in the econometric model, shown in Appendix A indicate that, on average, there is evidence supporting the validity of the parallel trends assumption. The estimated coefficients for the treatment and control groups demonstrate similar pre-treatment trends, suggesting that any difference between treated and controlled municipalities after the implementation of SWC will not result from pre-existing differences between municipalities enrolled in a solid waste public consortium.

Models that incorporate covariates exhibit superior performance in the parallel trends hypothesis test compared to models that do not include these variables. When there is any statistical significance in the pre-treatment period, as observed in some models (such as charts 146 and 147), this significance typically manifests within a span of one to two years, with the results not substantially deviating from a null effect.

**Fig. 3:**
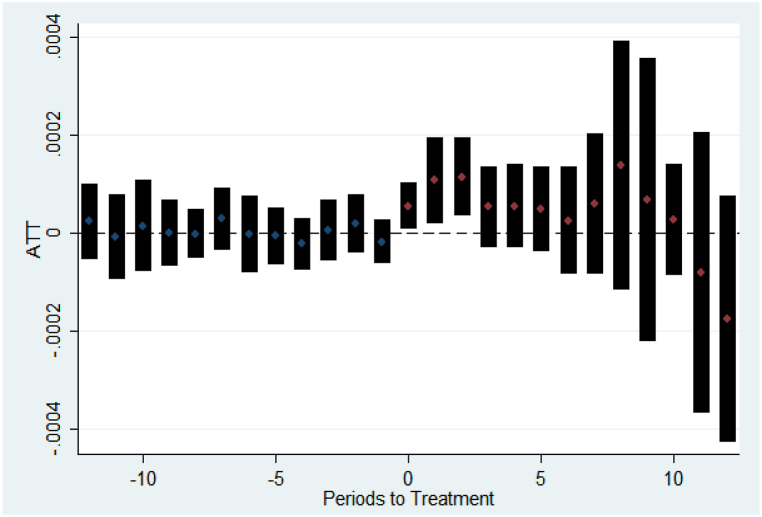
Sample of parallel trends test - Diarrhea/gastroenteritis – Hospitalizations (30–39 years).

Source: elaborated by authors.

A sample of the parallel trends test is shown in Fig. 3, highlighting that the average treatment effect on the treated (ATT) is centered around zero before the treatment. Thus, it indicates the reliability of the Staggered DID estimator employed in the analysis.

Table 4 presents a comparison of the results derived from the staggered DID models, providing the ATT results clustered by municipalities and SWC, both with and without covariates. Columns (1) and (2) do not use covariates, while covariates are included in columns (3) and (4). Models (1) and (3) use the municipality cluster, whereas models (2) and (4) use the SWC cluster.

**Table 4:** Cross-comparisons among models for significant outcomes Cross-comparisons among models for significant outcomes.

Models	Staggered DID			
	(1)	(2)	(3)	(4)
Incidence of non-standard fecal coliforms	0.450872**	0.450872*	0.304497	0.304497
PC - Approved procedures	−0.023182	−0.023182	−0.031804	−0.031804
MC - Approved procedures	0.01980	0.019810	0.000898	0.000898
HC - Approved procedures	0.104612**	0.104612	−0.029379	−0.029379
Schistosomesis Notifiable diseases	−0.00014**	−0.00014**	−0.000156**	−0.000156**
Hepatitis Notifiable diseases	0.000022	0.000022	0.000017	0.000017
Leptospirosis Notifiable diseases	0.000002	0.000002	0.000000	0.000000
Dengue Notifiable diseases	−0.000720	−0.000720	−0.000565	−0.000565
Acute Chagas disease Notifiable diseases	−0.000128	−0.000128	−0.000603	−0.000603
Diarrhea/gastroenteritis – Hospitalizations (less 5 years)	−0.000114**	−0.000114*	−0.000127**	−0.000127**
Diarrhea/gastroenteritis - Hospitalizations(5–9 years)	0.000045*	0.000045*	0.000066**	0.000066**
Diarrhea/gastroenteritis - Hospitalizations(10–14 years)	0.000025	0.000025	0.000052**	0.000052**
Diarrhea/gastroenteritis - Hospitalizations(15–19 years)	0.000007	0.000007	0.000032	0.000032
Diarrhea/gastroenteritis - Hospitalizations(30–39 years)	0.00007**	0.00007**	0.000103**	0.000103**
Diarrhea/gastroenteritis - Hospitalizations(40–49 years)	0.000023	0.000023	0.000045*	0.000045**
Other intestinal diseases - Hospitalizations(5–9 years)	−0.000034	−0.000034	−0.000019	−0.000019
Other intestinal diseases - Hospitalizations(10–14 years)	−0.00007**	−0.00007*	−0.000084**	−0.000084**
Other intestinal diseases - Hospitalizations(15–19 years)	0.558284	0.557066	0.862025	0.862807
Other intestinal diseases - Hospitalizations(60–69 years)	−0.000018	−0.000018	−0.000014	−0.000014
Other intestinal diseases - Hospitalizations(70–79 years)	−0.000028	−0.000028	−0.000012	−0.000012
Expenditure – Sanitation	5.642785	5.642785*	3.334580	3.334580
Expenditure – Environmental	−3.311307**	−3.311307**	−3.626831**	−3.626831**

Obs. The numbers with one star means 10% significance, while two stars means 5% confidence interval results. Columns (1) and (2) estimates do not use covariates, and the covariates are present in columns (3) and (4). Models (1) and (3) use the municipality cluster, while models (2) and (4) use the SWC cluster.

As it can be verified, clustered results by municipality/consortium are hardly different and show consistency when the municipality belongs to an SWC, indicating robustness in the results. Among the highlights are the indicators of Schistosomiasis, Diarrhea/gastroenteritis, Other intestinal diseases, and Environmental Expenditures. All significant results exhibit consistency in expected signals, and especially for diarrhea/gastroenteritis cases, show a strong causal relationship of SWC in reducing hospitalizations.

Overall, the results show that the participation of public consortia in the provision of solid waste services in Brazilian municipalities is relevant for improving some health-related indicators and reducing expenses in the environmental budget. The staggered DID showed that the significance level for results without covariates is slightly altered when comparing clusters. Another significant result is the alternate signal: the positive effect could be, in this case, interpreted as a negative outcome of SWM because water contamination rises in the period or results from the self-declared data.^3^

Regarding disease notifications, the most robust finding was for Schistosomiasis. The results significantly supported this, showing a consistent direction and magnitude of effect across various models (with covariates, the direction of the signals, clustered models, and even the magnitude of effects that remained consistent). Estimates indicate a significant decrease in reported cases, with a magnitude of −0.00014 in models 1 and 2 (without covariates) and −0.000156 in models 3 and 4 (with covariates) (both results are statistically significant at 5%). This demonstrates a consistent and robust effect of waste management policies in preventing Schistosomiasis. Conversely, for Hepatitis, Leptospirosis, and Acute Chagas, the results, including covariates, did not reveal significant findings.

Although the notification indicators showed significant results only for Schistosomiasis, the other models indicated that the accuracy of notification data might improve over time, as the few numbers of observations probably interfered with the estimations. This finding aligns with the diagnosis of Saiani, Mendonça, and Kuwahara [14], who noted similar challenges in evaluating solid waste policies using other health-related datasets.

In the case of “Dengue” notifications, the relevance of the outcome was discarded, as endemic vectors, such as the Aedes Aegypti mosquito,^4^ can be favored depending on the rain cycles (e.g., El Niño rain cycles), negligence of local governments to control mosquitoes during the summer, population leniency in avoiding the accumulation of water in their houses, among other factors [35,36]. Additionally, as warned by Benevenuto et al. [41], the results of sanitation indicators may require a more extended period of improvement to robustly affect notification data in municipalities.

For hospitalization data, on the one hand, for “Diarrhea/gastroenteritis Hospitalizations” the models were consistent but mixed results across multiple age ranges. In the age group up to 5 years, the models indicated a decrease (−0.000127 in models 3 and 4), corroborating the literature. However, other age groups exhibited significant increases in hospitalizations. In contrast, we observed the inverse results in the “Other intestinal diseases” hospitalizations, with only the 10–14 years being significant (−0.00007 in model 3 and -0,000084 in model 4). These results are aligned with the findings presented in prior studies by Saiani, Mendonça, and Kuwahara [14], and Epstein [22]. This suggests that the implementation of SWC initiatives can positively impact health outcomes, potentially leading to a reduction in hospitalizations related to cases of diarrhea and gastroenteritis within the targeted region.

When comparing the outcome variables evaluated in this study with other examples in the literature, the list shown in Table 4 appears to be exhaustive. For instance, Nascimento et al. [28] focused on the primary care strategy known as Family's Health,^5^ particularly in impoverished communities. However, as the data was discontinued in 2015, limiting our analysis to only ten years of policies, we believe it is not appropriate to use this information for our purposes. Besides some tests that have been done, we consider it better for further studies to evaluate more specific procedures of Primary Care Complexity, such as the variable “Fetal Growth and Malnutrition” – which, in this case, did not show significant results. This approach also applies to Medium and High-Complexity procedures, especially since air and water contamination are associated with various types of neoplasia ([22]; Saiani, Mendonça & Kuwahara, 2015; [25]).

Regarding the “Incidence of non-standard fecal coliforms^6^”, the estimations may reflect the “Lake Wobegon Effect” (Maxwell & Lopus, 1994) in the SNIS database, attributable to self-reported bias (Maxwell & Lopus, 1994; [37]). Firstly, the issue arises from the increasing reporting of previously missing information over time, as more municipalities participate, potentially introducing a positive bias in water contamination data. Secondly, the possibility of intentional errors being made to show apparent improvements over time could compromise the reliability of results for this specific indicator.to This type of conduct can invalidate the result for this specific indicator.

Finally, regarding the last indicators in Table 4, specifically the expenditure in the Sanitation and Environmental sector (3.63 in models 3 and 4 for environmental expenses), the findings indicate a reduction in reported values. This aligns with Cattani et al. [6], which suggests that basic technological waste treatment routes can guarantee better outcomes for the population. In terms of budgetary information, while the models did not provide conclusive evidence of a reduction in sanitation sector spending, they confirmed cost savings in environmental outcomes for treated municipalities. This supports the hypothesis that 10.13039/501100000042SWC can act as a local coordinator, enhancing both health and fiscal indicators for associated municipalities, as highlighted by Silvestre et al. [31].

The study's detailed results underscore the critical importance of implementing SWC and their substantial impact on public health and municipal finances. The direct and significant relation between the reduction of notifiable diseases (particularly Schistosomiasis) and waste management practices underscores the crucial role of SWCs. By coordinating and optimizing waste management, SWCs contribute significantly to reducing environments conducive to the proliferation of parasitic diseases and other pathogens. The notable decrease in Schistosomiasis cases reflects the success of environmental sanitation policies implemented alongside the consortia, proving efficient waste management is a powerful tool in combating these and other diseases.

Additionally, hospitalization data for Diarrhea/Gastroenteritis illustrate the direct effects of waste management policies on population health. The varied results across age groups point to the necessity for age-specific policies and practices. The reduction in hospitalization rates in children under 5 years is especially relevant, indicating that SWC policies effectively protect vulnerable groups and reduce diseases linked to poor waste management.

Financially, SWCs play a crucial role in optimizing costs related to sanitation and environmental management. The trend of cost reduction suggests that the consortia's treatment and disposal methods are both efficient and economical. This is vital for budget-constrained allowing resource reallocation to other essential areas like health and education. Moreover, the economic efficiency of SWCs can encourage more municipalities to join such consortia, enhancing public health and environmental sustainability.

In conclusion, this study demonstrates the significance and effectiveness of Solid Waste Consortia in promoting public health and optimizing municipal finances. The reduction of notifiable diseases, decrease in hospitalization rates, and economic efficiency are clear indicators of the positive impact of SWCs. They foster healthier and safer environments and promote sustainable, cost-effective resource management, underscoring the need to continue and expand these initiatives.

## 5 Final considerations and policy implications

The Brazilian legal framework for solid waste policy has been strongly enhanced recently. PLANARES significantly contributes to long-term national strategic planning by elucidating the challenges associated with biological risks inherent in improper waste disposal. It serves as a practical guide for operationalizing the legal provisions, principles, objectives, and guidelines outlined in the National Solid Waste Policy (PNRS, 2010). The PLANARES framework offers a coherent rationale for transitioning from illicit and incorrect waste disposal practices to legally sanctioned and environmentally sound methods. This Plan provides not only a diagnostic of the SWM sector in the country but also examines national and international scenarios and macroeconomic trends. Based on these insights, several goals, guidelines, projects, programs, and actions are included in the National Plan, aiming to achieve the objectives of the PNRS over a 20-year horizon (up to 2040) (UNDP & ME, 2022b).

In addition to the PNRS and PLANARES, another critical policy instrument is Law No. 14,026/2020, the New Legal Framework for Basic Sanitation (Novo Marco do Saneamento Básico, NMSB)[51]. This law amends Federal Law No. 11,445/2007, introducing incentives for the regionalized provision of services by the private sector, leveraging gains of scale, and accelerating the universalization of SWM services. The recent policy update offers two main benefits: (i) it removes barriers to private sector participation in these services, and; (ii) the enhances the public consortia law (Law No. 11,107/2005) and its incentives for management joint public services [51].

Nevertheless, despite the Brazilian government's recent effort to establish and dictate guidelines for the solid waste sector, estimating the causality between variables and their societal impacts remains challenging. While studies such as Saiani [23] and Benevenuto et al. (2022) highlight the benefits of increased private sector participation in the sanitation infrastructure, the impact of scaling up the number of public consortia operating SWM services remained underexplored in the academic literature. In this sense, the main contribution of this pioneer study is to technically demonstrate the causality among the proper policy for solid waste i.e. PC, and their health and budgetary outcomes in Brazil.

Considering these factors, we estimated models for various health indicators: water quality, approved procedures categorized by complexity, notifications by epidemiology and morbidity classification, hospitalizations by epidemiology, morbidity, and age ranges classifications, and level of expenditures in environmental and sanitation. We obtained robust results for health indicators, specifically hospitalizations due to Schistosomiasis, Diarrhea/gastroenteritis (in children up to 5 years of age), and other intestinal diseases (for the 10–14 years of age).

In the case of hospitalization data, we achieved a notable result in the 0–5 years age range, showing a negative and significant outcome. This indicates that SWC can improve public health by reducing the number of hospitalizations due to Diarrhea/gastroenteritis in their acting region. However, for other age ranges, we found robust results with positive effects, which could challenge the findings for the 0–5 years age group. These results may be influenced by Brazil's demographic age distribution, which is skewed towards older age groups, and by other uncontrolled diseases that can cause diarrhea symptoms by multiple reasons. In future studies, it will be prudent to control for these positive effects with additional covariates to ensure accurate treatment effects.

Regarding budgetary information, although the reduction in sanitation expenses was not confirmed in the models, the environmental results from this robust technique did confirm a reduction in expenses for the treated municipalities. This aligns with the exploratory research of Cattani et al. [6] and supports the notion that SWC can serve as a local coordinator, improving both health and fiscal indicators for municipalities participating in PCs.

Concerning the study's limitations, one is the restricted analysis of other outcomes such as economic growth, climate change, and human development. Further research and discussions on these indicators are necessary to comprehensively address outcomes and improve the databases used for robust econometric modeling. Regarding notification data, the diseases analyzed require more investigation to clarify the transmission types of pathogen transmission and their vectors (mechanical, biological, etc.). Additionally, considering the potential of missing SNIS dataset information, future studies might benefit from examining water quality using alternative indexes or datasets that more directly reflect water and sewage treatment interventions. Lastly, the lack of sectoral activity data limited our analysis of important controlled groups like waste pickers, as detailed in the study by Cruvinel et al. [38].

A transversal policy recommendation emerging from this research is to enhance the self-reported data available through DATASUS. The differences in the results from the eight tested models underscore the need for careful interpretation of the self-declared data to avoid biased conclusions. Furthermore, fostering collaboration among relevant stakeholders, including environmental agencies, health departments, and waste management authorities, is crucial for ensuring consistent and accurate data reporting. Efforts to improve data accessibility and transparency, allow researchers to conduct more robust analyses that can serve as evidence to policymakers.

Regarding the solid waste data, recent improvements by the Federal Government, such as the recently launched SINIR database, are noteworthy. However, the utility of panel methods, which require more frequent data observations,^7^ may be limited for future studies. Distinguishing between administrative data (such as approved procedures and hospitalizations) and disease notifications is also important for future research. Since some diseases (such as those listed in this study) may not be included in administrative data due to symptom treatment at home, notification datasets could provide more accurate and timely health-related data. A limitation of this study is the inability to temporally assess the adequacy of solid waste disposal, as data before 2015 are unreliable. Therefore, our focus was on comparing public consortia for urban solid waste management against stand-alone municipal operations, examining their impact on health indicators and budgetary expenses.

Nevertheless, this ex-post study has demonstrated how publicly available data can be effectively used to provide solid evidence-based through econometric models to guide solid waste policymaking. Despite data challenges and the nascent stage of SWC in Brazil, the results point out significant benefits from promoting greater cooperation among municipalities. The findings clearly show that regional planning and the current implementation of solid waste policies done by public consortia can positively impact the population's health and lead to more rationality in public spending.

## Funding

This study was financed in part by the 10.13039/501100002322Coordenação de Aperfeiçoamento de Pessoal de Nível Superior – Brasil (CAPES) – Finance Code 001.

## CRediT authorship contribution statement

**Yan Nonato Cattani:** Writing – original draft, Supervision, Methodology, Investigation, Funding acquisition, Formal analysis, Conceptualization. **Raquel Pereira Pontes:** Visualization, Validation, Software, Formal analysis, Data curation. **Diego Camargo Botassio:** Validation, Software, Formal analysis, Data curation. **Daniel Komesu:** Visualization, Validation, Software, Formal analysis, Data curation. **Rodolfo Gomes Benevenuto:** Writing – review & editing. **Mario Henrique Ogasavara:** Writing – review & editing, Writing – original draft.

## Declaration of competing interest

The authors declare that they have no known competing financial interests or personal relationships that could have appeared to influence the work reported in this paper.

## Footnotes

1 A newly developed method for estimating Staggered Difference-in-Differences, designed for cases where units remain treated in all periods following treatment, was introduced by Sun and Abraham (2021). The primary distinction between this and the method by Callaway and Sant'Anna (2021) lies in the estimation approach. Sun and Abraham (2021) utilize linear regressions for their estimates, whereas Callaway and Sant'Anna (2021) offer three distinct estimation techniques: (i) regression, (ii) inverse probability weighting, and (iii) doubly robust estimation. In our study, we chose to employ the first estimation method proposed by Callaway and Sant'Anna [39].

2 According to the most essential document of the public consortia initiative, the Intention Protocol, the activities of public consortia must be specified to provide such services. It is usual to determine all types of solid waste activities to avoid legal constraints, even though the consortia only use a few.

3 The self declared data issues are detailed below within the description of the “Lake Wobegon Effect”.

4 The Aedes Aegypti can spread dengue and other diseases such as chikungunya, zika, and yellow fever.

5 In Portuguese, “ESF – Estratégia Saúde da Família" is a national policy that establishes a medical team composed of multidisciplinary profiles, such as generalist doctors, nurses, technicians, and dentists, among other health professionals, to treat poor communities throughout Brazil.

6 Morita et al. [3] points out coliforms as one emerging contaminant in irregular dumpsites.

7 The verified information is dated only from 2015 to 2019 in SINIR.
