# Supplementary material for: “Measuring the health and fiscal outcomes of solid waste management operations by intergovernmental arrangements: The case of public consortia in Brazil”
Source: Heliyon. 2024 Feb 18;10(4):e26032. doi: 10.1016/j.heliyon.2024.e26032 (PMC10906158; doi:10.1016/j.heliyon.2024.e26032)
Supplement: Multimedia component 1 [file mmc1.docx]

# Appendix A: Parallel Test

Without Covariates


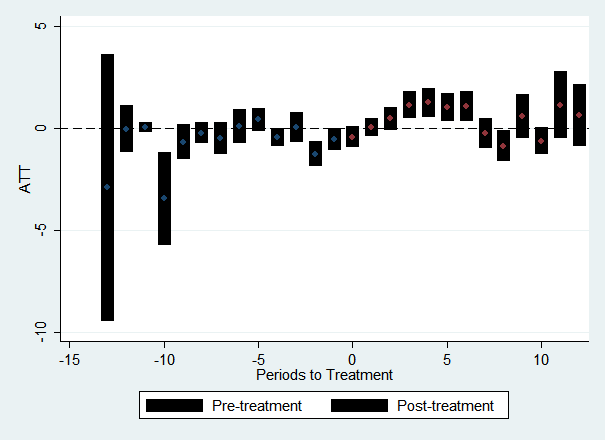


Chart 1 – Y1 - Incidence Of Non-Standard Fecal Coliforms - Municipal Cluster


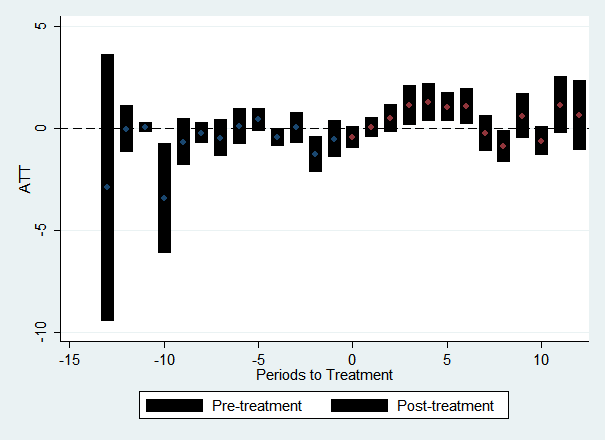


Chart 2 – Y1 - Incidence of non-standard fecal coliforms - consortia cluster
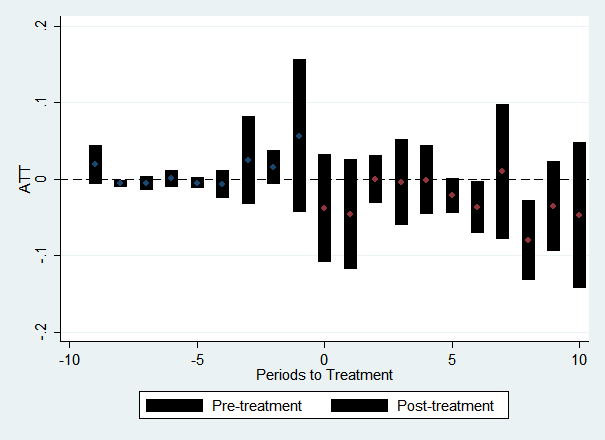


Chart 3 – Y2 - PC - Approved procedures - municipal cluster

.......

.......................................................

....
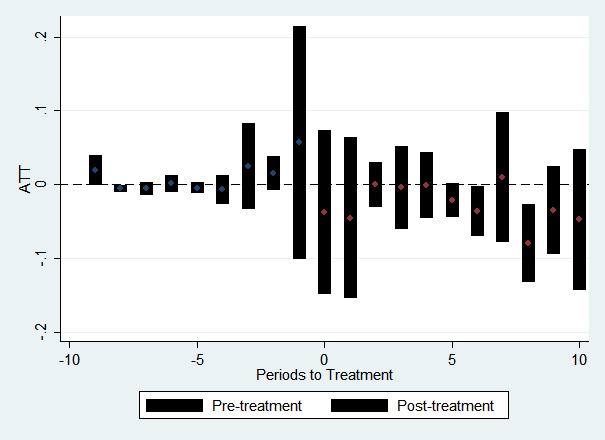


Chart 4 – Y2 - PC - Approved procedures - consortia cluster


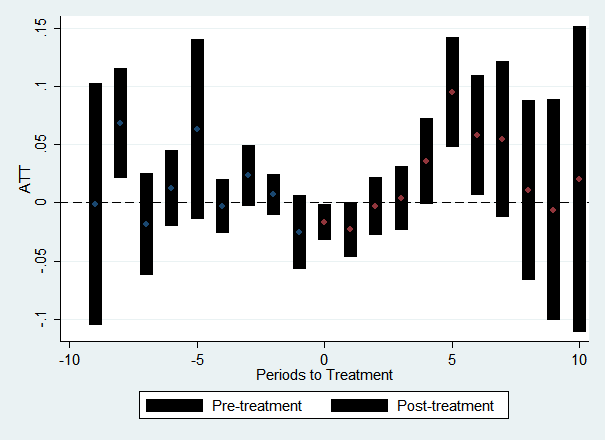


Chart 5 – Y3 - MC - Approved procedures - municipal cluster


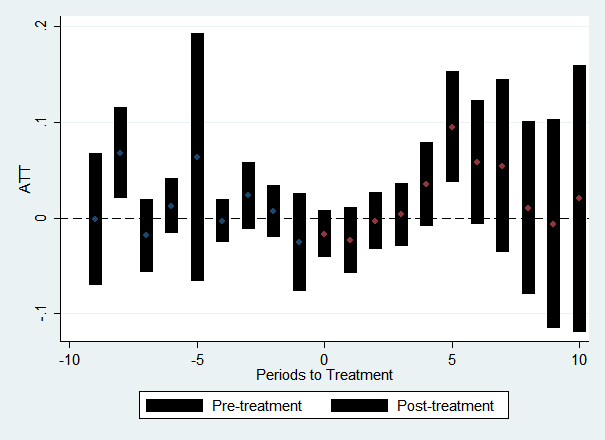


Chart 6 – Y3 - MC - Approved procedures - consortia cluster


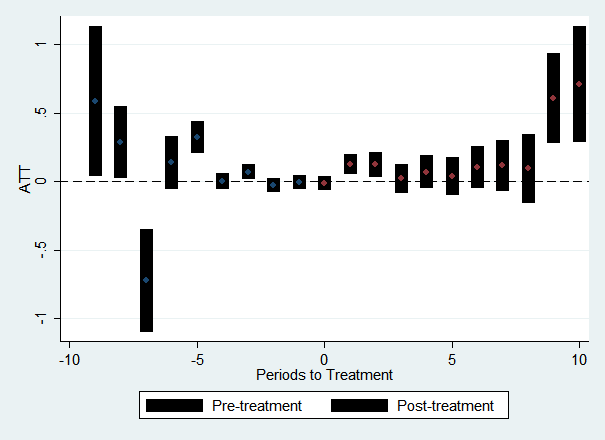


Chart 7 – Y4 - HC - Approved procedures - municipal cluster


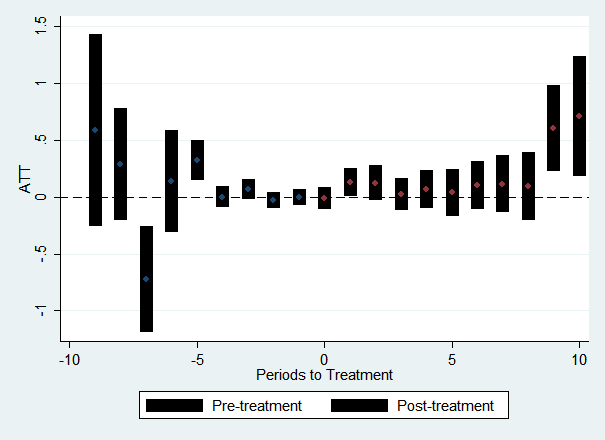


Chart 8 – Y4 - HC - Approved procedures - consortia cluster


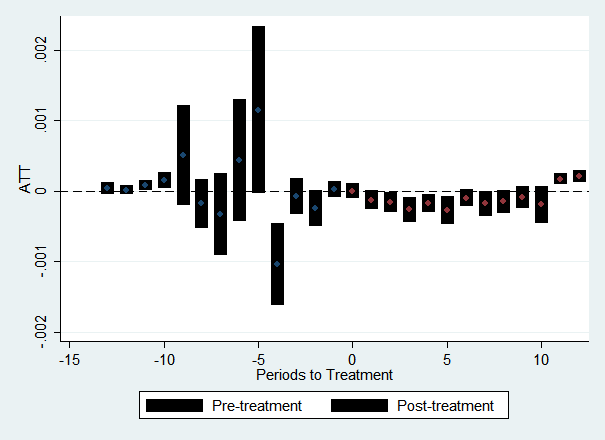


Chart 9 – Y5 - Schistosomiasis Notifiable diseases - municipal cluster


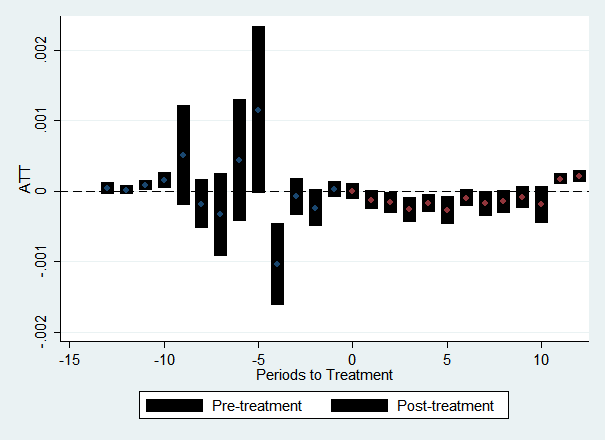


Chart 10 – Y5 - Schistosomiasis Notifiable diseases - consortia cluster
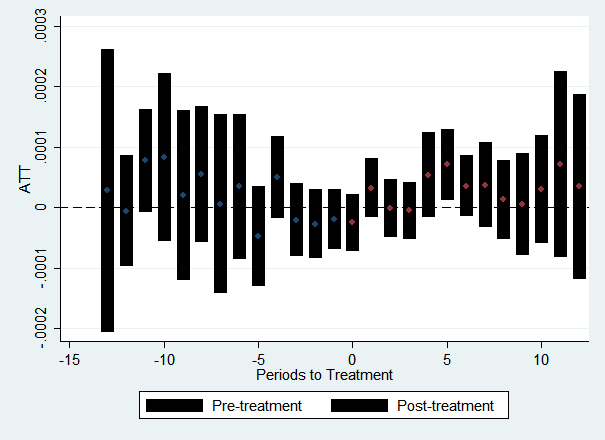


Chart 11 – Y6 - Hepatitis Notifiable diseases - municipal cluster


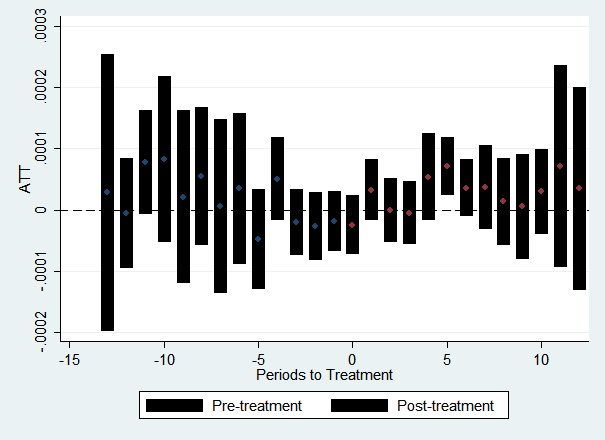


Chart 12 – Y6 - Hepatitis Notifiable diseases - consortia cluster


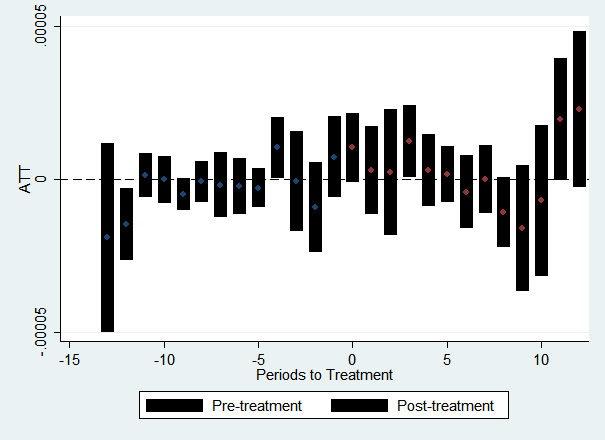


Chart 13 – Y7 - Leptospirosis Notifiable diseases - municipal cluster


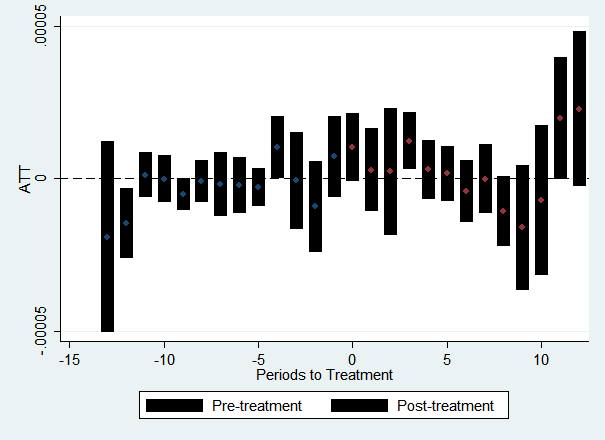


Chart 14 – Y7 - Leptospirosis Notifiable diseases - consortia cluster


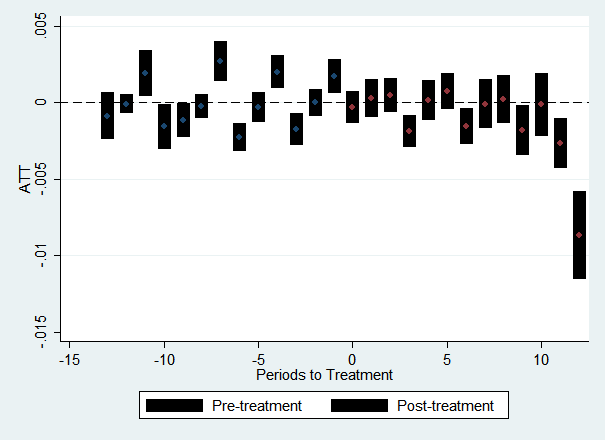


Chart 15 – Y8 - Dengue Notifiable diseases - municipal cluster


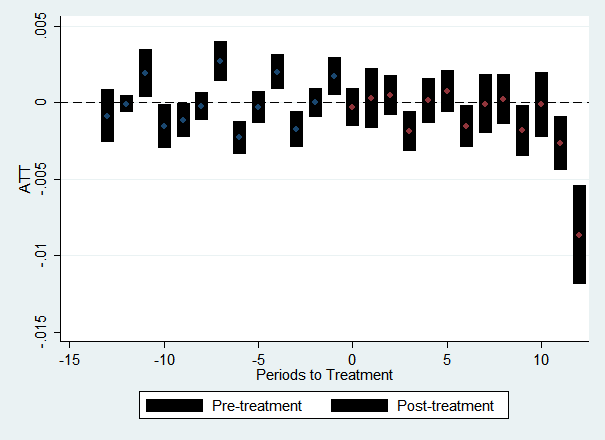


Chart 16 – Y8 - Dengue Notifiable diseases - consortia cluster


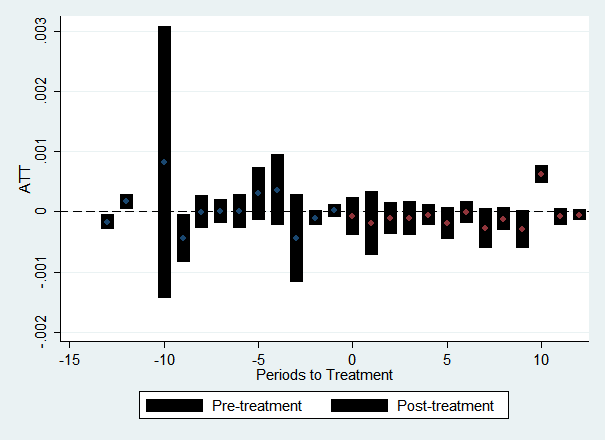


Chart 17 – Y9 - Acute Chagas disease Notifiable diseases - municipal cluster


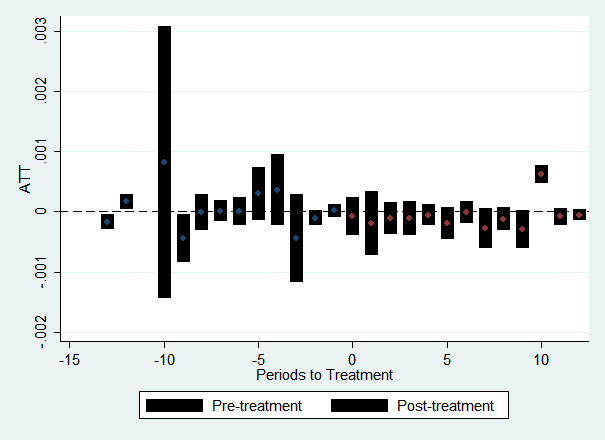


Chart 18 – Y9 - Acute Chagas disease Notifiable diseases - consortia cluster


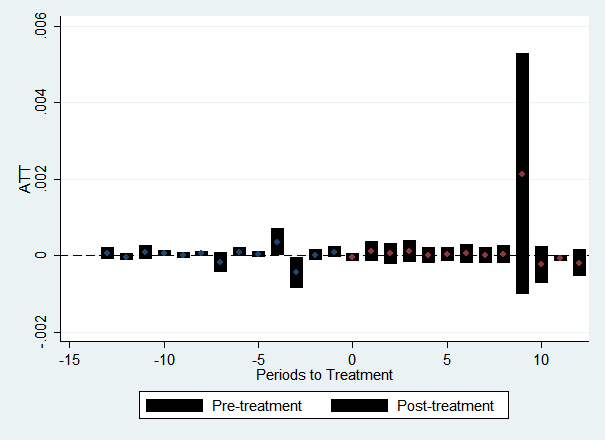


Chart 19 – Y10 - Malaria Notifiable diseases - municipal cluster


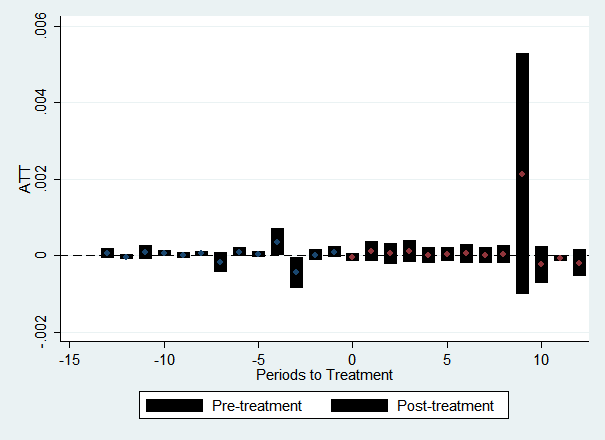


Chart 20 – Y10 - Malaria Notifiable diseases - consortia cluster


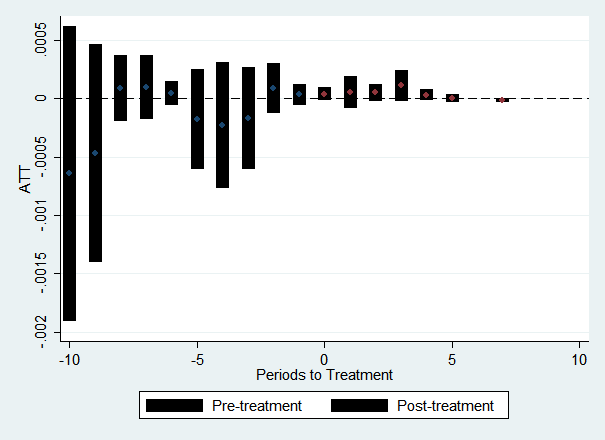


Chart 21 – Y11 - Dermatosis Notifiable diseases - municipal cluster


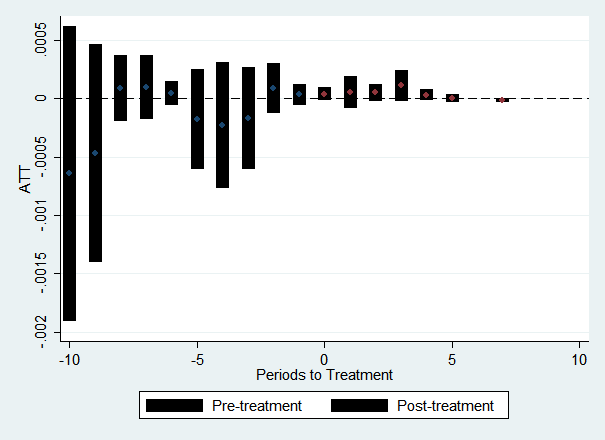


Chart 22 – Y11 - Dermatosis Notifiable diseases - consortia cluster


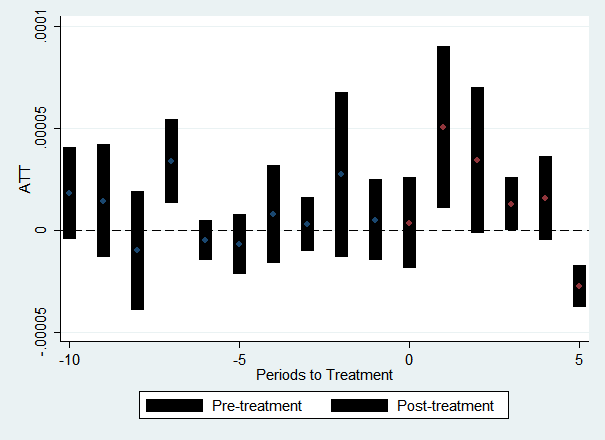


Chart 23 – Y12 - Yellow fever Notifiable diseases - municipal cluster


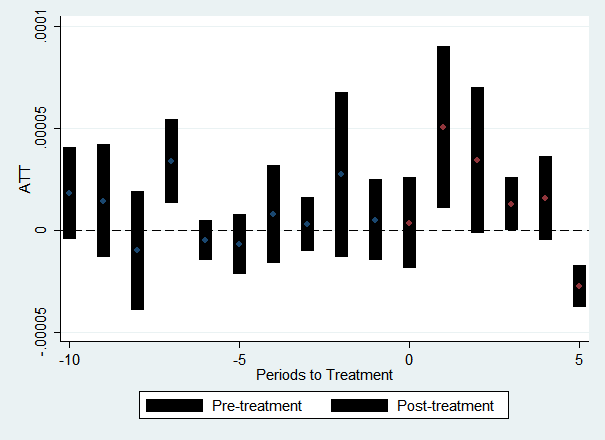


Chart 24 – Y12 - Yellow fever Notifiable diseases - consortia cluster


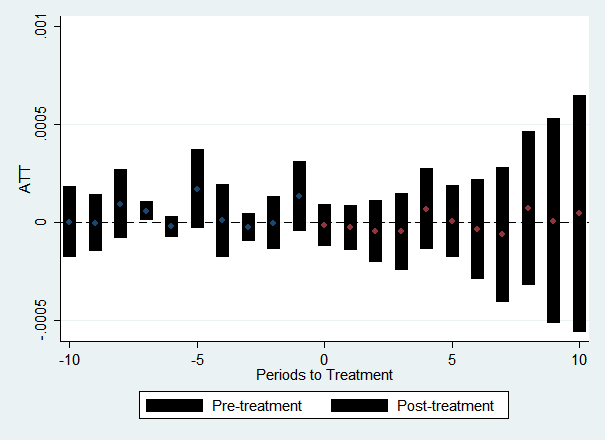


Chart 25 – Y13 - Hantavirus Notifiable diseases - municipal cluster


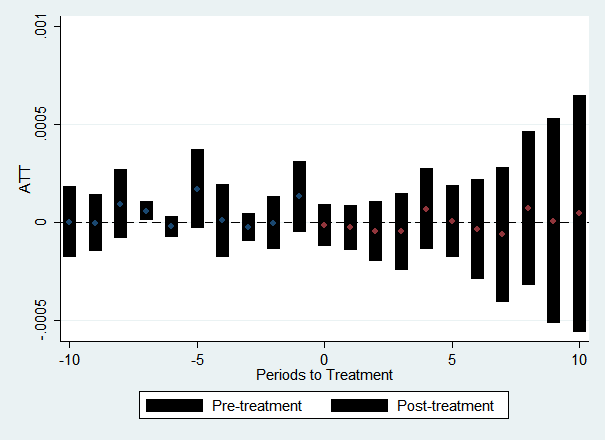


Chart 26 – Y13 - Hantavirus Notifiable diseases - consortia cluster


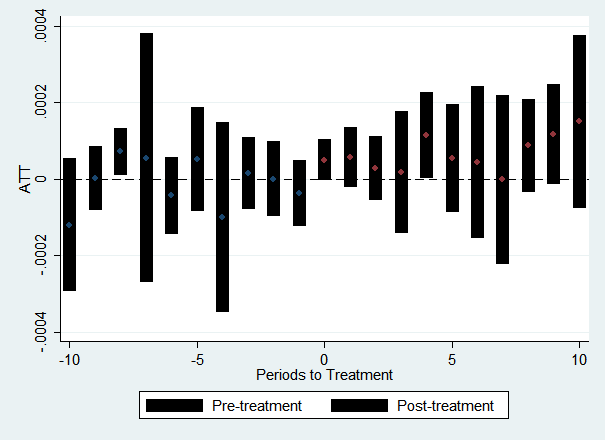


Chart 27 – Y14 - Leishmaniasis Notifiable diseases - municipal cluster


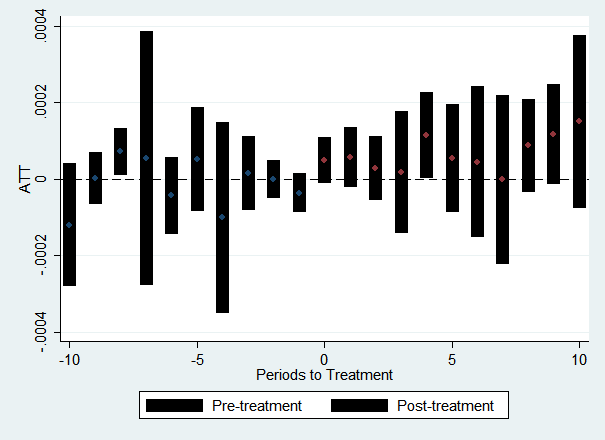


Chart 28 – Y14 - Leishmaniasis Notifiable diseases - consortia cluster


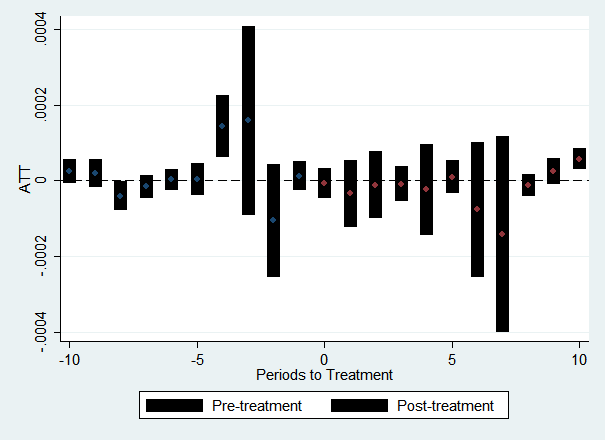


Chart 29 – Y15 - Whooping Notifiable diseases - municipal cluster


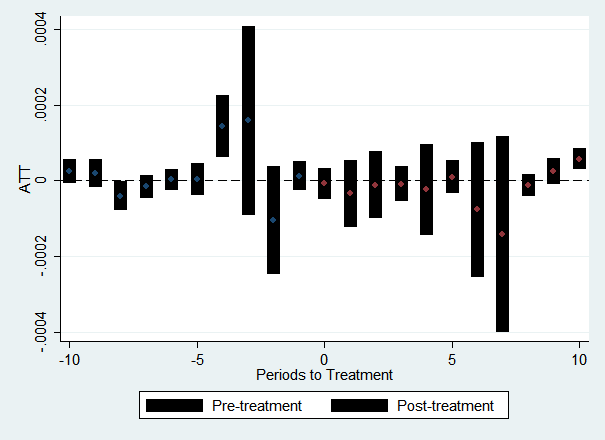


Chart 30 – Y15 - Whooping Notifiable diseases - consortia cluster


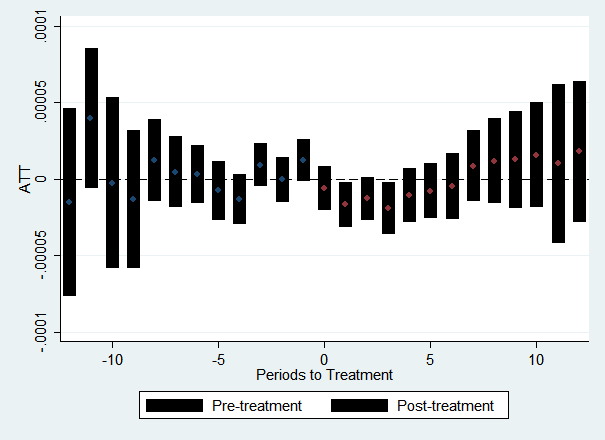


Chart 31 – Y16 - Fetal Growth and Malnutrition - Hospitalizations - municipal cluster


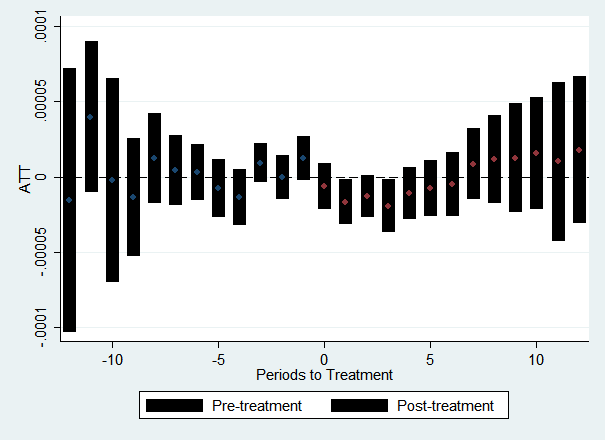


Chart 32 – Y16 - Fetal Growth and Malnutrition - Hospitalizations - consortia cluster


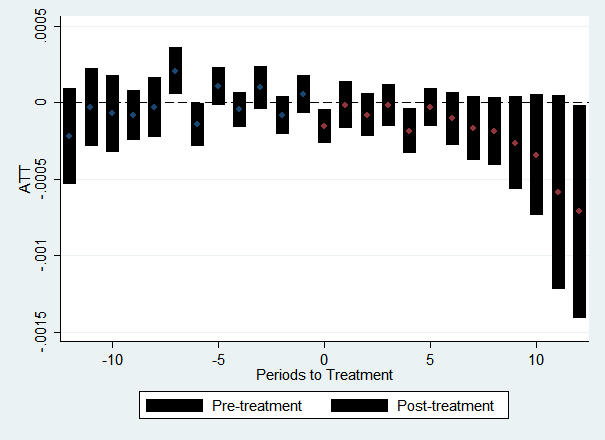


Chart 33 – Y17 - Diarrhea/gastroenteritis - Hospitalizations(less 5 years) - municipal cluster


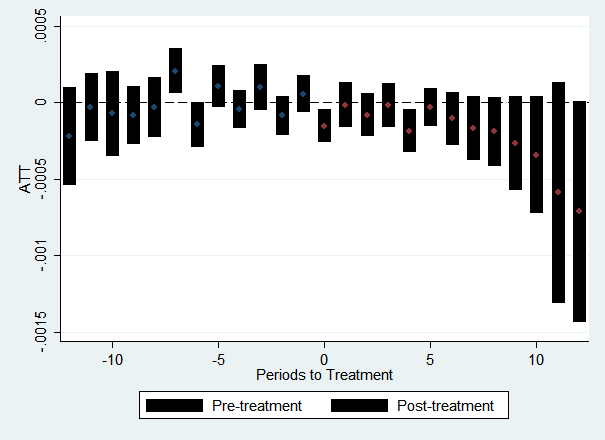


Chart 34 – Y17 - Diarrhea/gastroenteritis - Hospitalizations(less 5 years) - consortia cluster


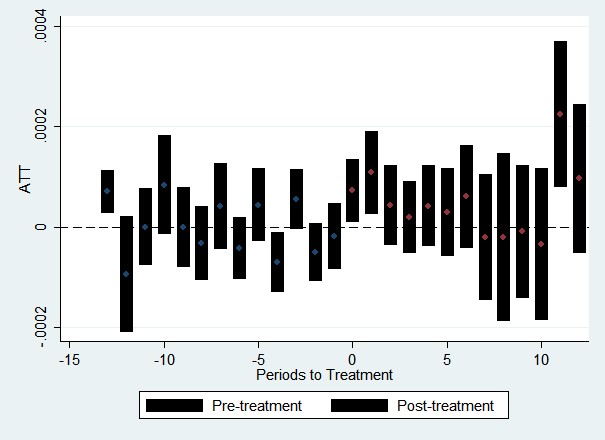


Chart 35 – Y18 - Diarrhea/gastroenteritis - Hospitalizations(5-9 years) - municipal cluster


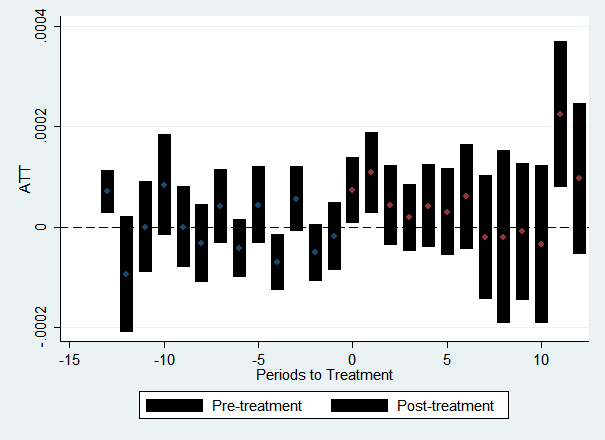


Chart 36 – Y18 - Diarrhea/gastroenteritis - Hospitalizations(5-9 years) - consortia cluster


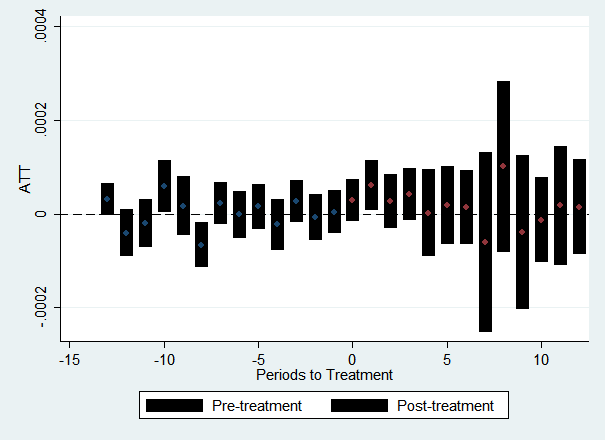


Chart 37 – Y19 - Diarrhea/gastroenteritis - Hospitalizations(10-14 years) - municipal cluster


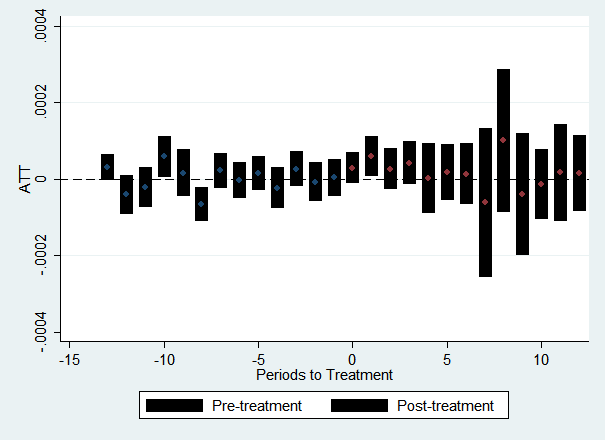


Chart 38 – Y19 - Diarrhea/gastroenteritis - Hospitalizations(10-14 years) - consortia cluster


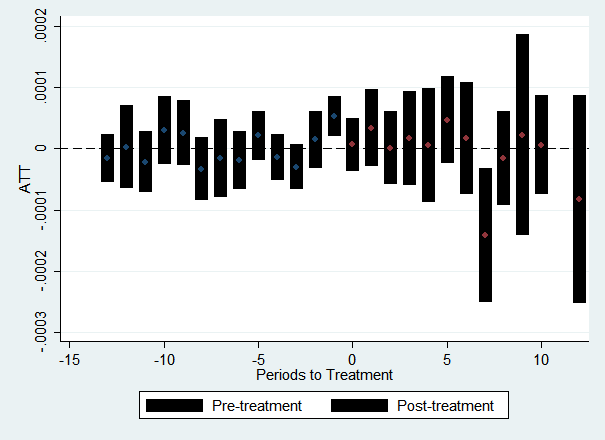


Chart 39 – Y20 - Diarrhea/gastroenteritis - Hospitalizations(15-19 years) - municipal cluster


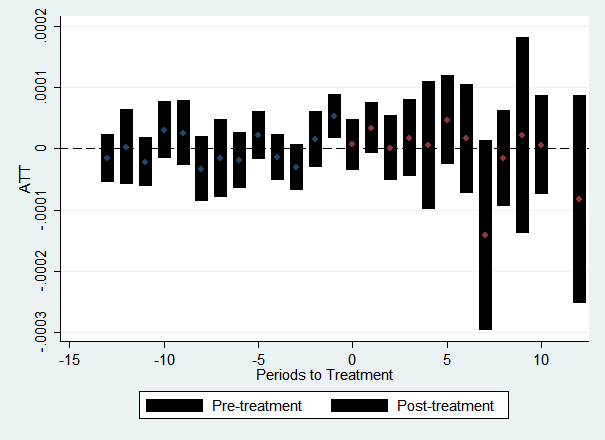


Chart 40 – Y20 - Diarrhea/gastroenteritis - Hospitalizations(15-19 years) - consortia cluster


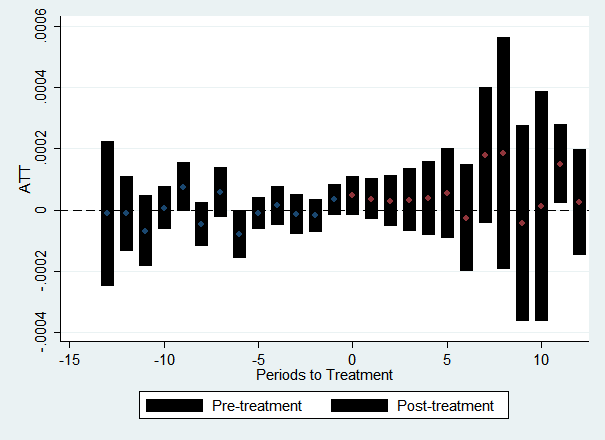


Chart 41 – Y21 - Diarrhea/gastroenteritis - Hospitalizations(20-29 years) - municipal cluster


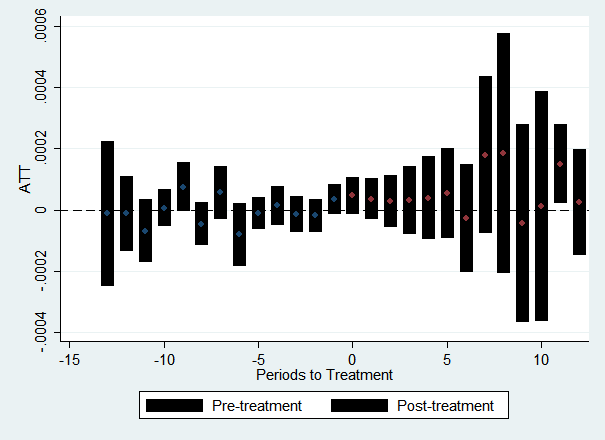


Chart 42 – Y21 - Diarrhea/gastroenteritis - Hospitalizations(20-29 years) - consortia cluster


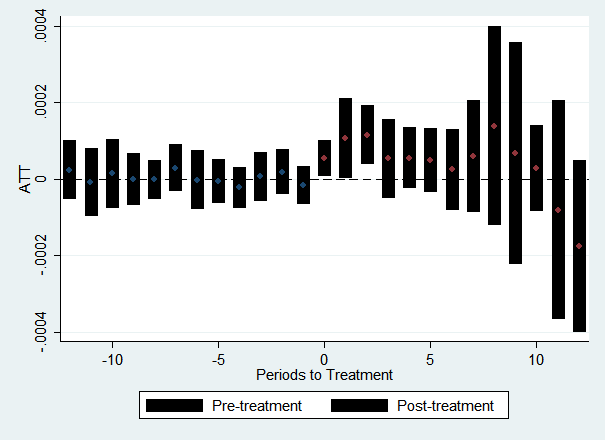


Chart 43 – Y22 - Diarrhea/gastroenteritis - Hospitalizations(30-39 years) - municipal cluster


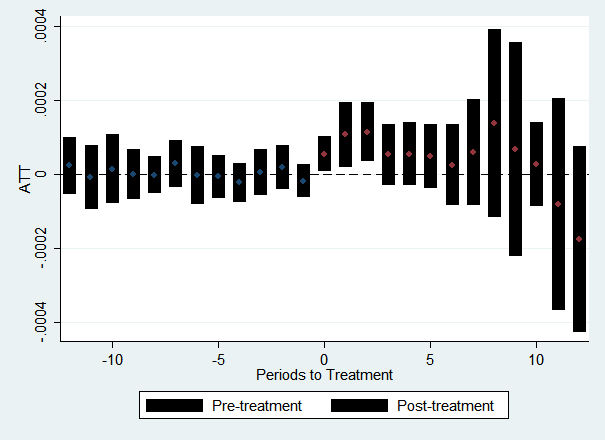


Chart 44 – Y22 - Diarrhea/gastroenteritis - Hospitalizations(30-39 years) - consortia cluster


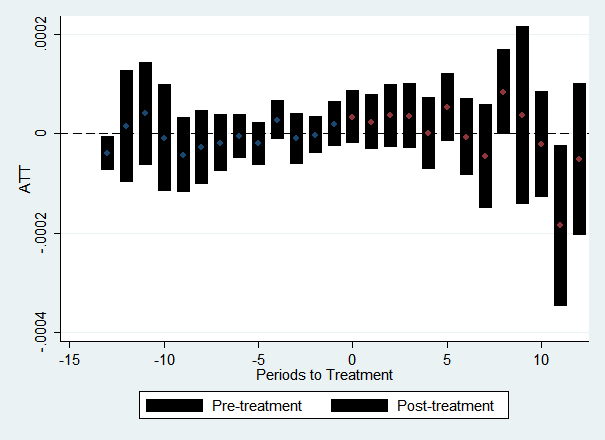


Chart 45 – Y23 - Diarrhea/gastroenteritis - Hospitalizations(40-49 years) - municipal cluster


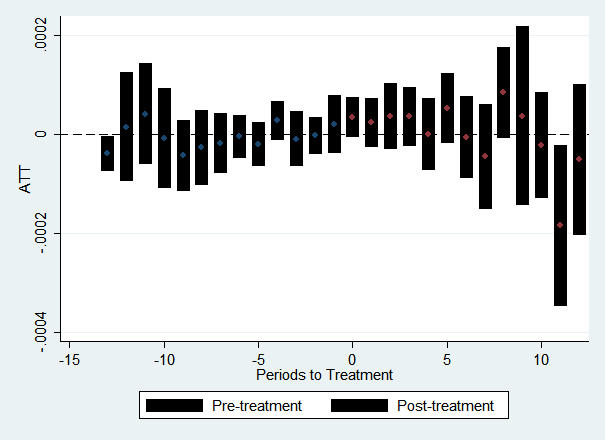


Chart 46 – Y23 - Diarrhea/gastroenteritis - Hospitalizations(40-49 years) - consortia cluster


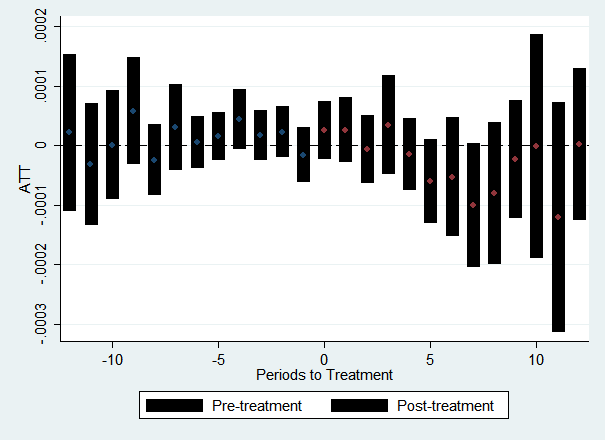


Chart 47 – Y24 - Diarrhea/gastroenteritis - Hospitalizations(50-59 years) - municipal cluster


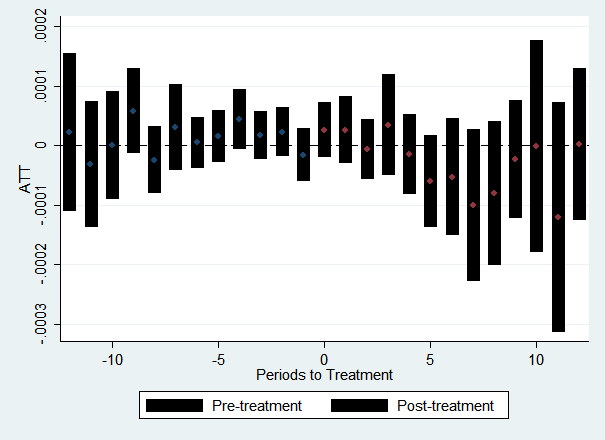


Chart 48 – Y24 - Diarrhea/gastroenteritis - Hospitalizations(50-59 years) - consortia cluster


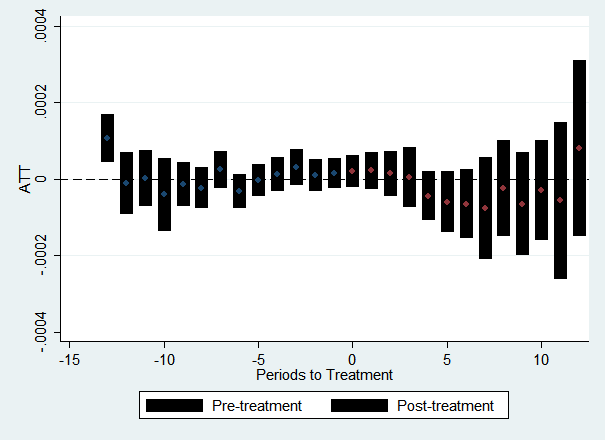


Chart 49 – Y25 - Diarrhea/gastroenteritis - Hospitalizations(60-69 years) - municipal cluster


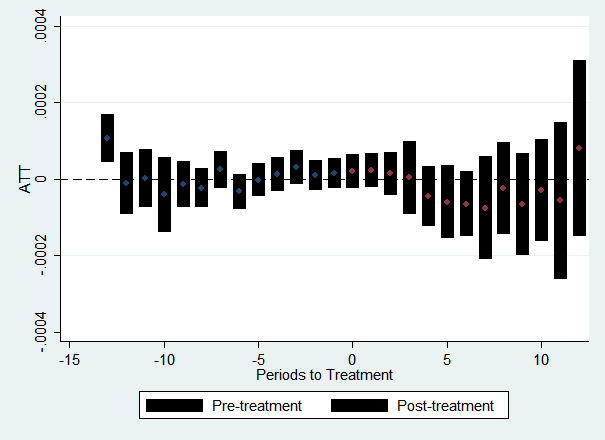


Chart 50 – Y25 - Diarrhea/gastroenteritis - Hospitalizations(60-69 years) - consortia cluster


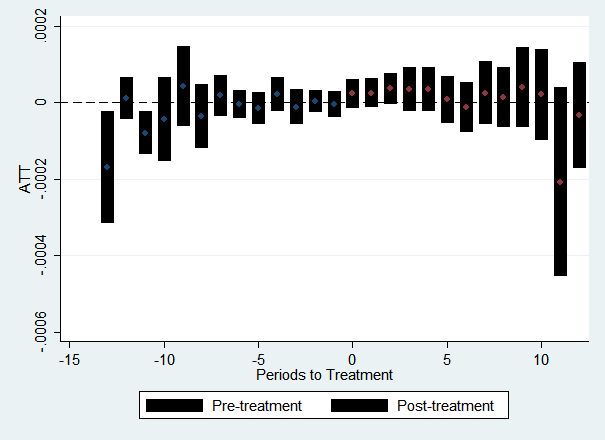


Chart 51 – Y26 - Diarrhea/gastroenteritis - Hospitalizations(70-79 years) - municipal cluster


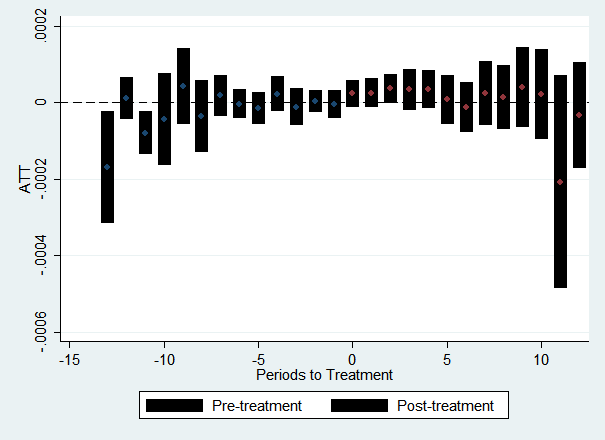


Chart 52 – Y26 - Diarrhea/gastroenteritis - Hospitalizations(70-79 years) - consortia cluster


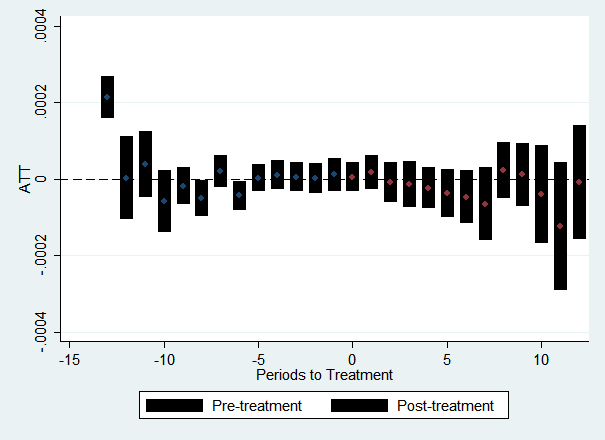


Chart 53 – Y27 - Diarrhea/gastroenteritis - Hospitalizations(+80 years) - municipal cluster


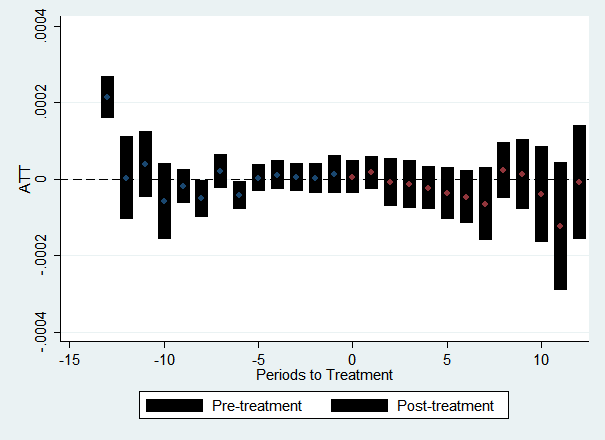


Chart 54 – Y27 - Diarrhea/gastroenteritis - Hospitalizations(+80 years) - consortia cluster


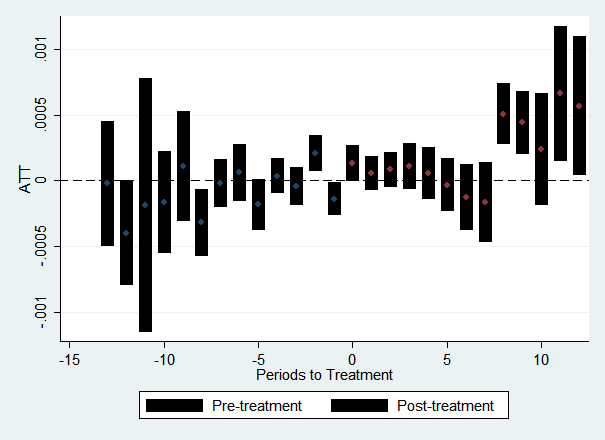


Chart 55 – Y28 - Other intestinal diseases - Hospitalizations(less 5 years) - municipal cluster


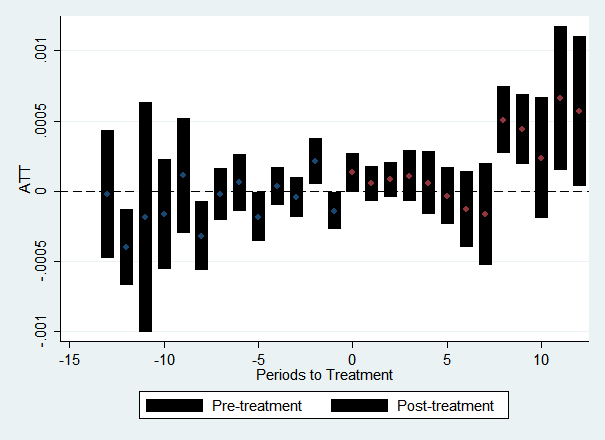


Chart 56 – Y28 - Other intestinal diseases - Hospitalizations(less 5 years) - consortia cluster


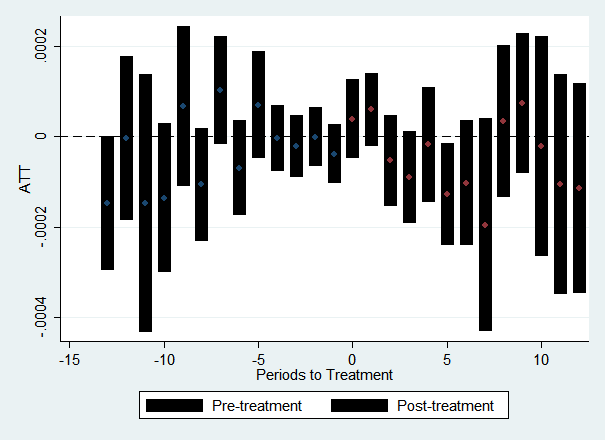


Chart 57 – Y29 - Other intestinal diseases - Hospitalizations(5-9 years) - municipal cluster


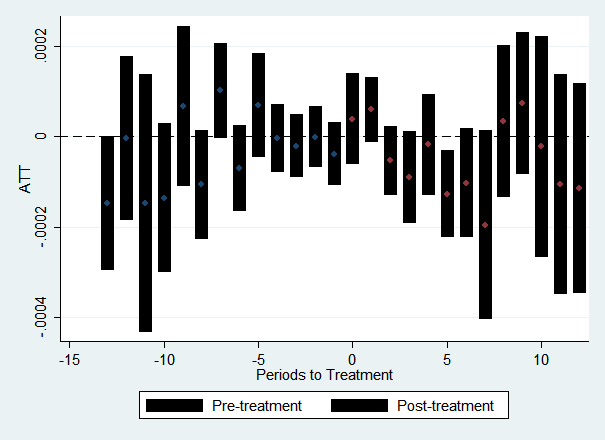


Chart 58 – Y29 - Other intestinal diseases - Hospitalizations(5-9 years) - consortia cluster


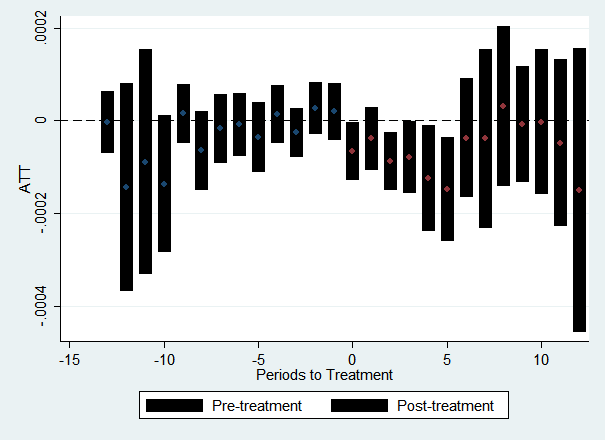


Chart 59 – Y30 - Other intestinal diseases - Hospitalizations(10-14 years) - municipal cluster


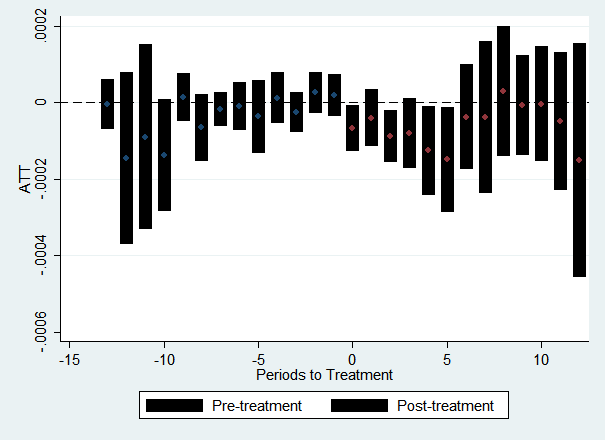


Chart 60 – Y30 - Other intestinal diseases - Hospitalizations(10-14 years) - consortia cluster


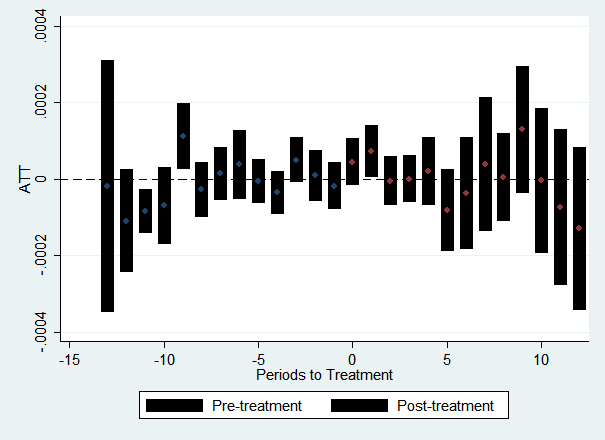


Chart 61 – Y31 - Other intestinal diseases - Hospitalizations(15-19 years) - municipal cluster


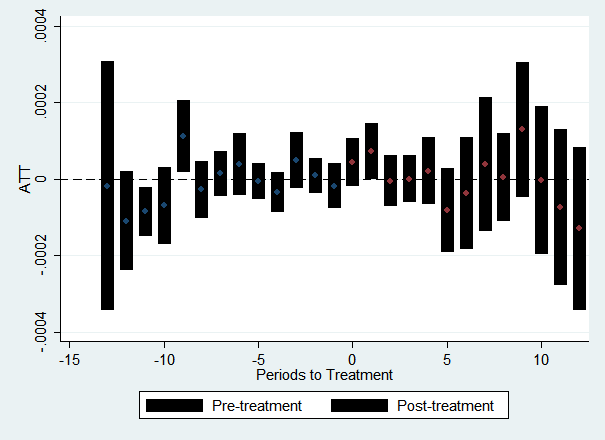


Chart 62 – Y31 - Other intestinal diseases - Hospitalizations(15-19 years) - consortia cluster


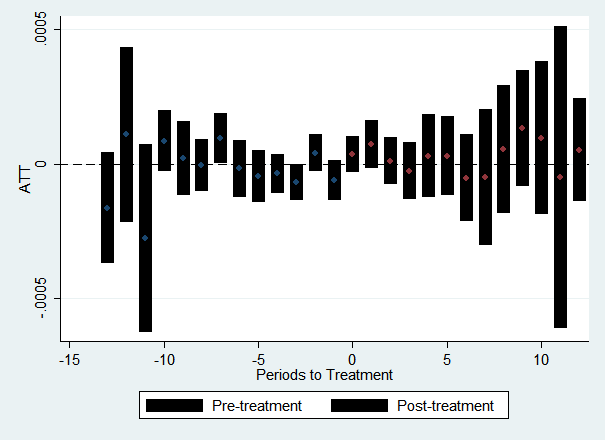


Chart 63 – Y32 - Other intestinal diseases - Hospitalizations(20-29 years) - municipal cluster


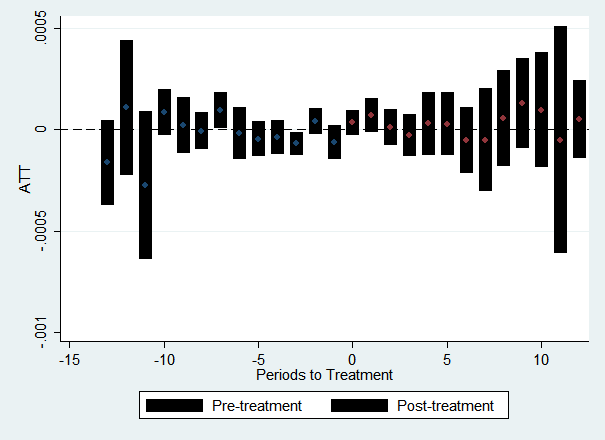


Chart 64 – Y32 - Other intestinal diseases - Hospitalizations(20-29 years) - consortia cluster


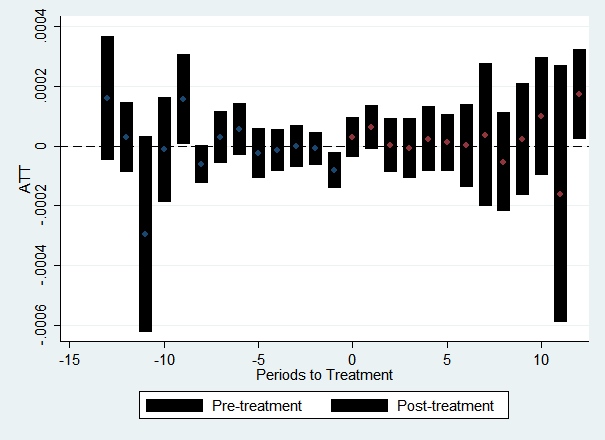


Chart 65 – Y33 - Other intestinal diseases - Hospitalizations(30-39 years) - municipal cluster


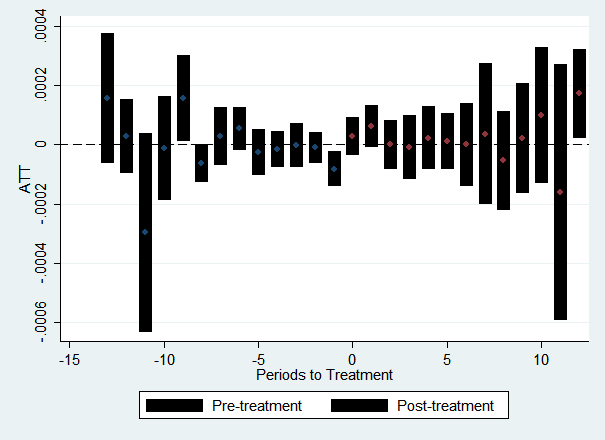


Chart 66 – Y33 - Other intestinal diseases - Hospitalizations(30-39 years) - consortia cluster


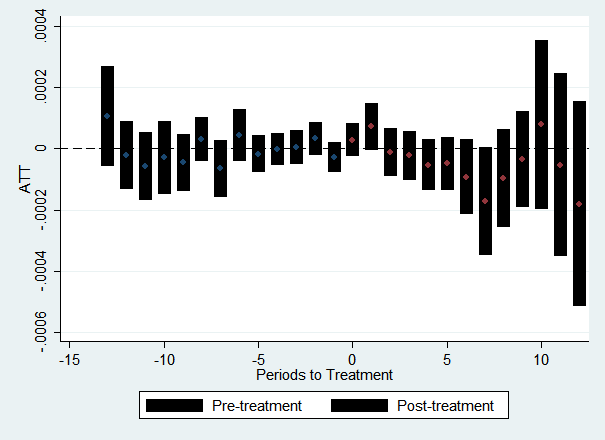


Chart 67 – Y34 - Other intestinal diseases - Hospitalizations(40-49 years) - municipal cluster


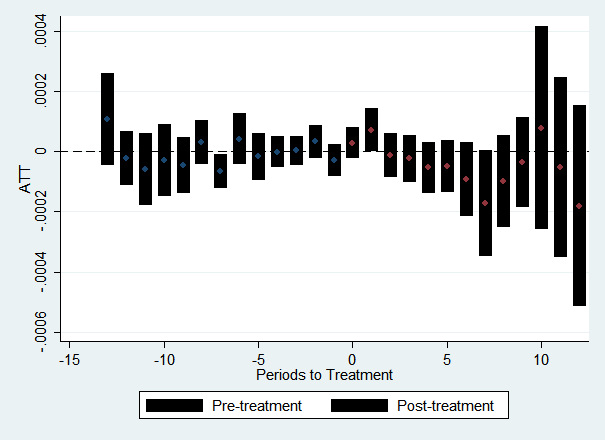


Chart 68 – Y34 - Other intestinal diseases - Hospitalizations(40-49 years) - consortia cluster


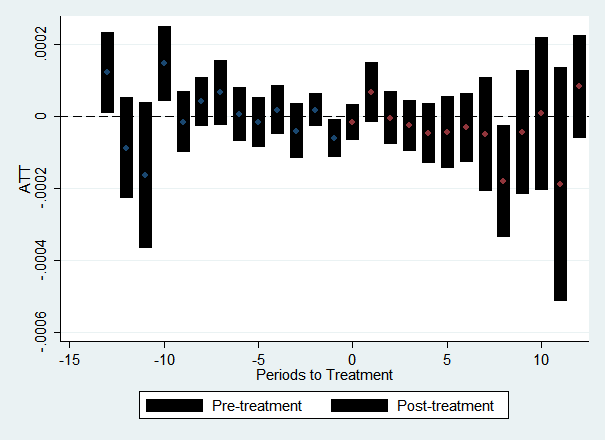


Chart 69 – Y35 - Other intestinal diseases - Hospitalizations(50-59 years) - municipal cluster


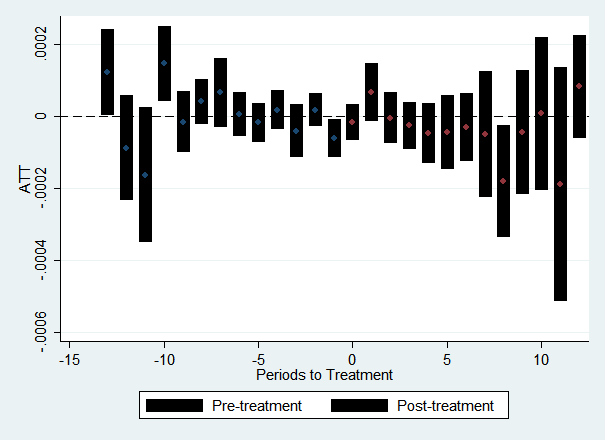


Chart 70 – Y35 - Other intestinal diseases - Hospitalizations(50-59 years) - consortia cluster


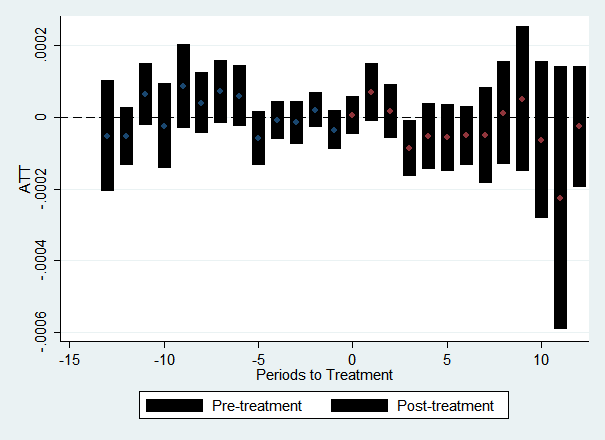


Chart 71 – Y36 - Other intestinal diseases - Hospitalizations(60-69 years) - municipal cluster


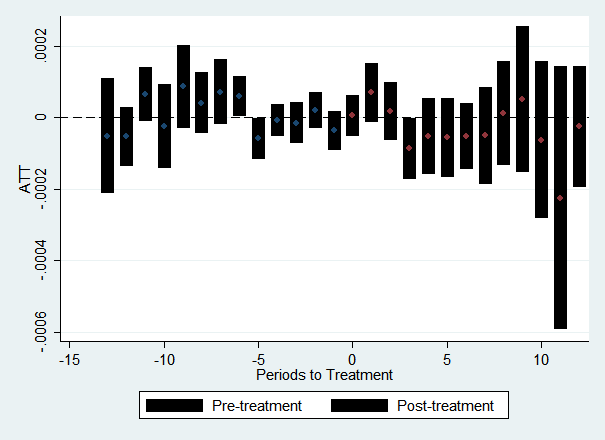


Chart 72 – Y36 - Other intestinal diseases - Hospitalizations(60-69 years) - consortia cluster


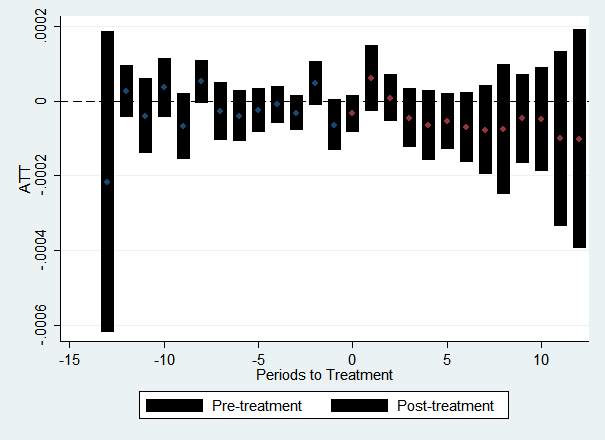


Chart 73 – Y37 - Other intestinal diseases - Hospitalizations(70-79 years) - municipal cluster


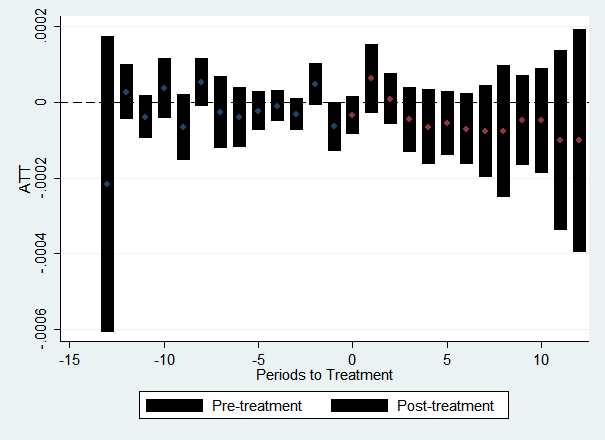


Chart 74 – Y37 - Other intestinal diseases - Hospitalizations(70-79 years) - consortia cluster


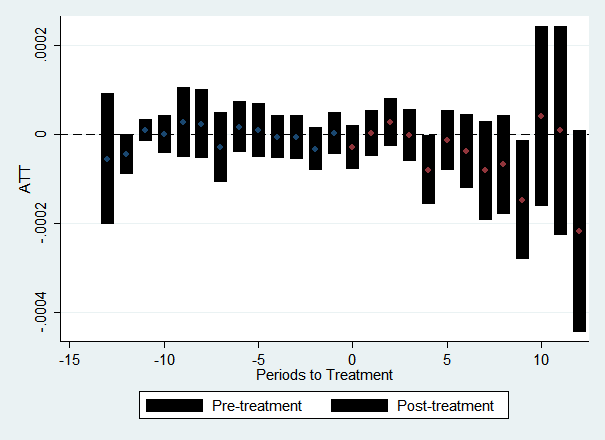


Chart 75 – Y38 - Other intestinal diseases - Hospitalizations(+80 years) - municipal cluster


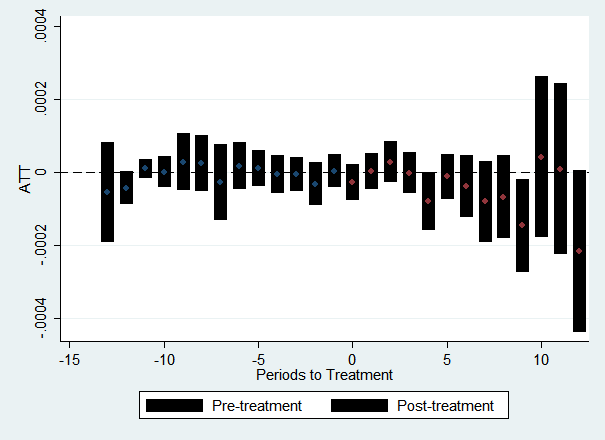


Chart 76 – Y38 - Other intestinal diseases - Hospitalizations(+80 years) - consortia cluster


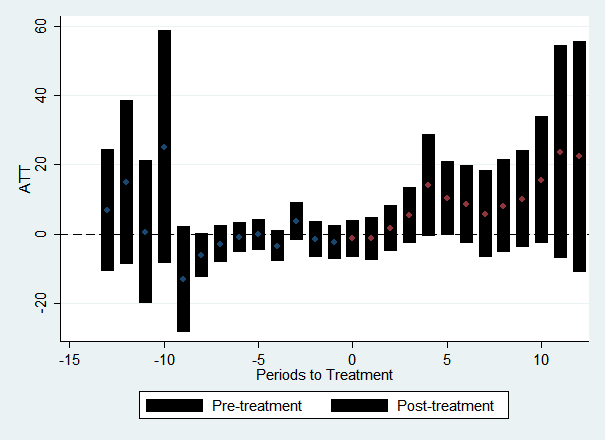


Chart 77 – Y39 - Expenditures - Sanitation - municipal cluster


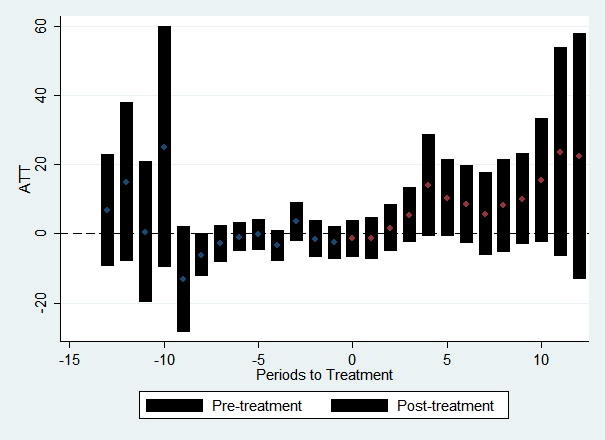


Chart 78 – Y39 - Expenditures - Sanitation - consortia cluster


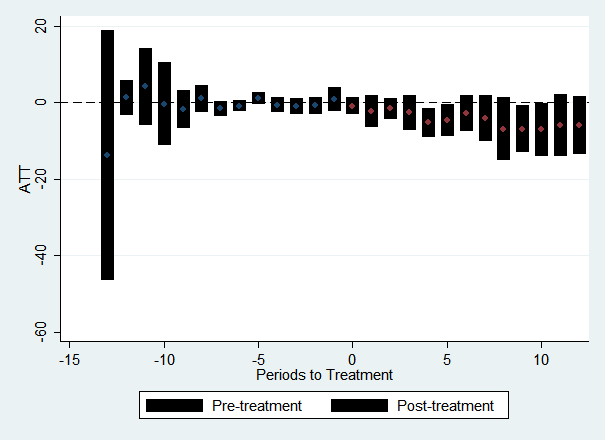


Chart 79 – Y40 - Expenditures - Environmental - municipal cluster


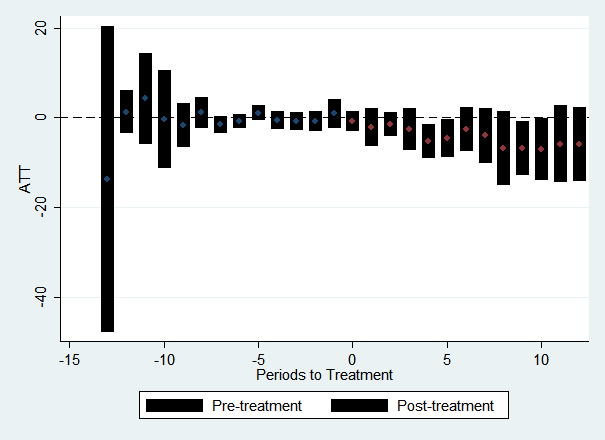


Chart 80 – Y40 - Expenditures - Environmental - consortia cluster

With Covariates


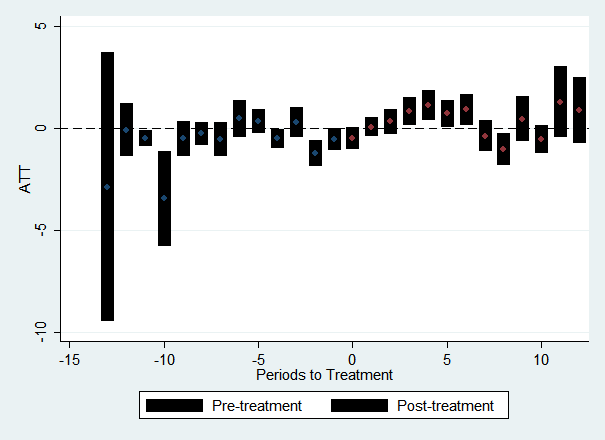


Chart 81 – With covariates: Y1 - Incidence of non-standard fecal coliforms - municipal cluster


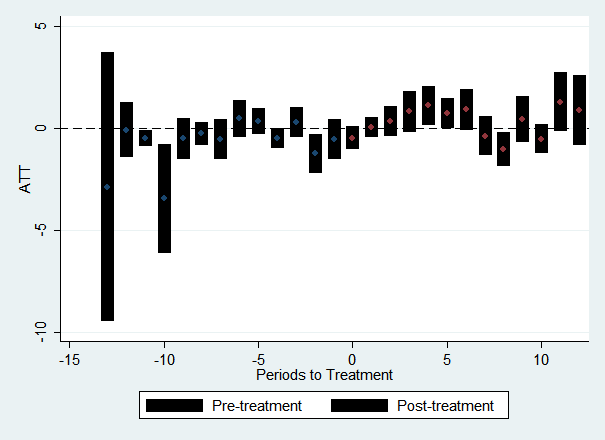


Chart 82 – With covariates: Y1 - Incidence of non-standard fecal coliforms - consortia cluster


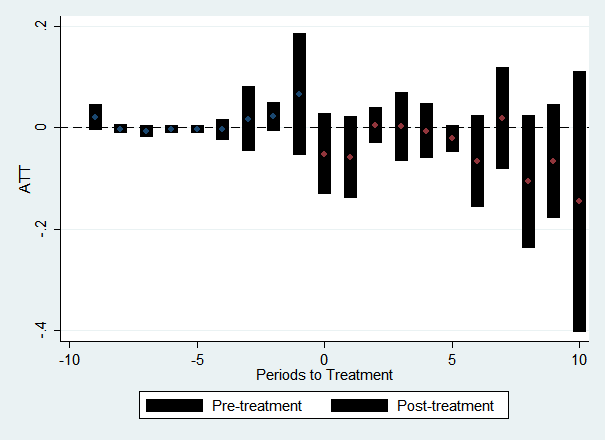


Chart 83 – With covariates: Y2 - PC - Approved procedures - municipal cluster

....
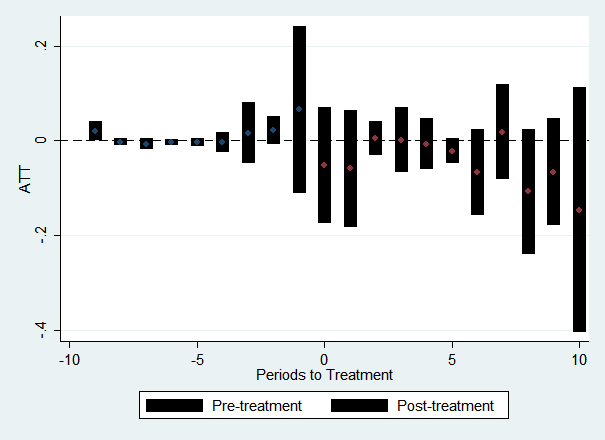


Chart 84 – With covariates: Y2 - PC - Approved procedures - consortia cluster


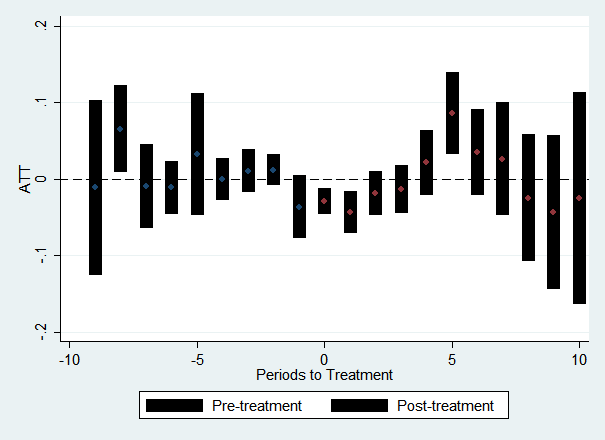


Chart 85 – With covariates: Y3 - MC - Approved procedures - municipal cluster


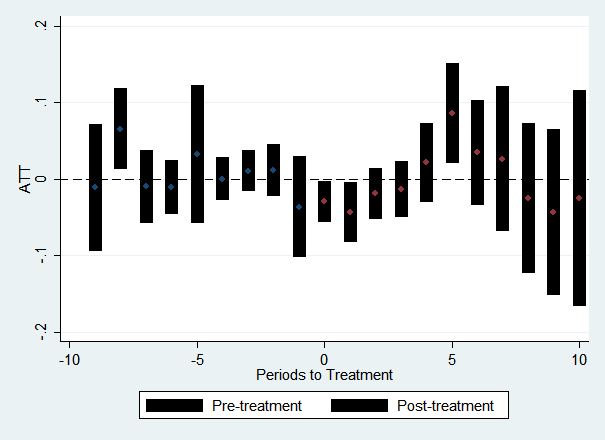


Chart 86 – With covariates: Y3 - MC - Approved procedures - consortia cluster


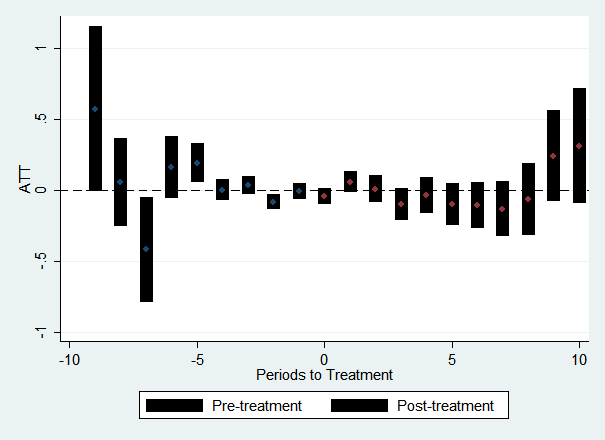


Chart 87 – With covariates: Y4 - HC - Approved procedures - municipal cluster


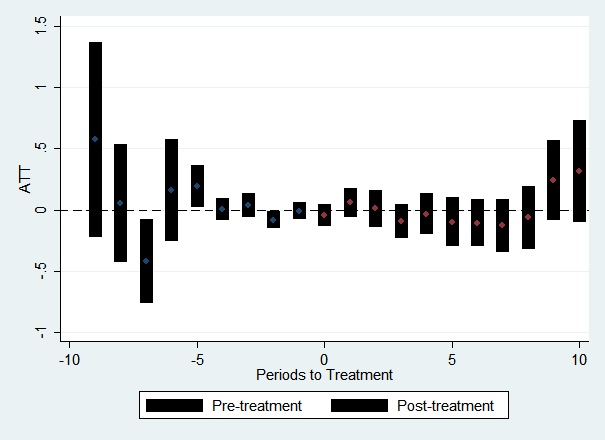


Chart 88 – With covariates: Y4 - HC - Approved procedures - consortia cluster


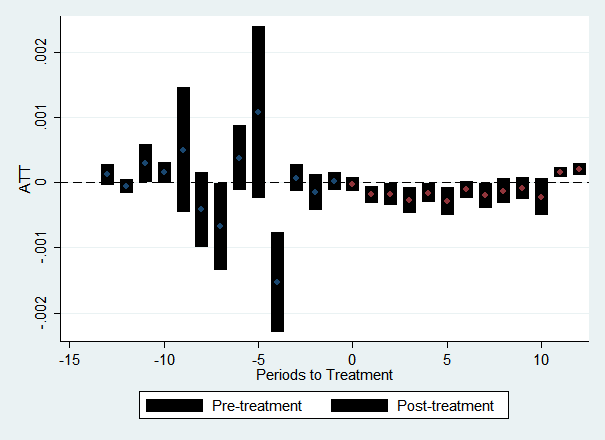


Chart 89 – With covariates: Y5 - Schistosomiasis Notifiable diseases - municipal cluster


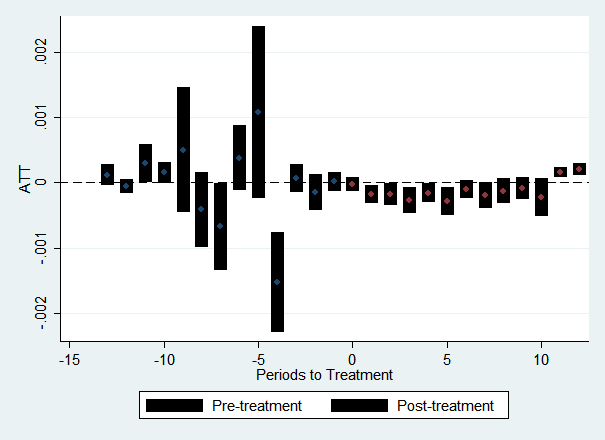


Chart 90 – With covariates: Y5 - Schistosomiasis Notifiable diseases - consortia cluster


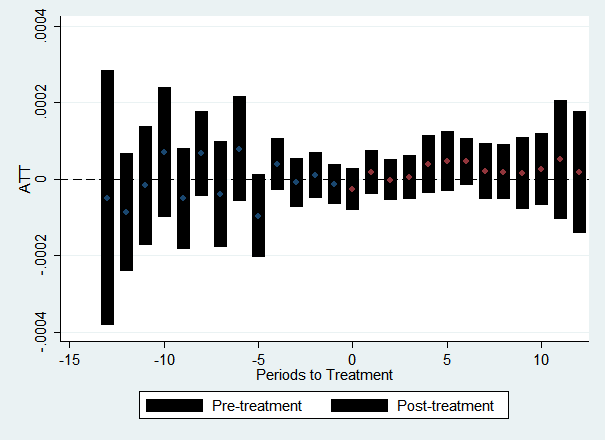


Chart 91 – With covariates: Y6 - Hepatitis Notifiable diseases - municipal cluster


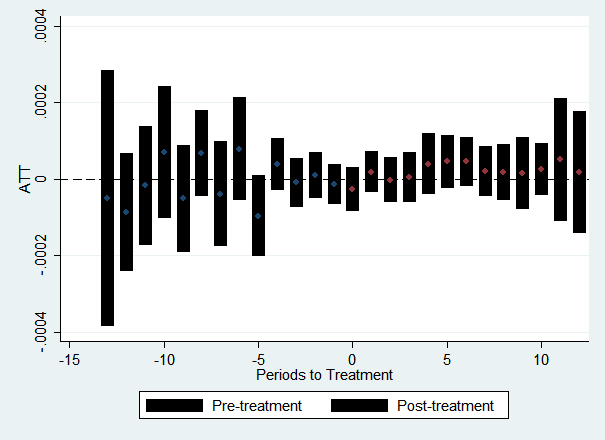


Chart 92 – With covariates: Y6 - Hepatitis Notifiable diseases - consortia cluster


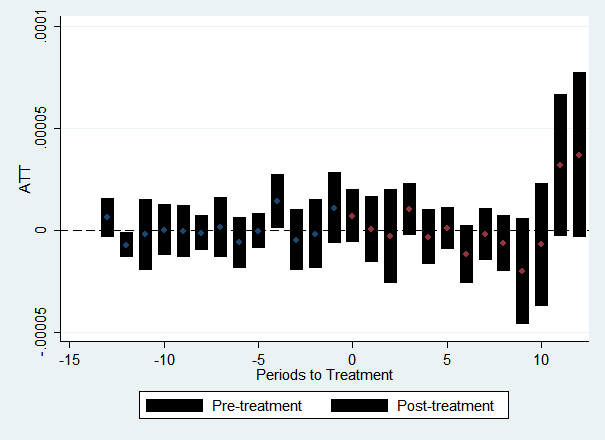


Chart 93 – With covariates: Y7 - Leptospirosis Notifiable diseases - municipal cluster


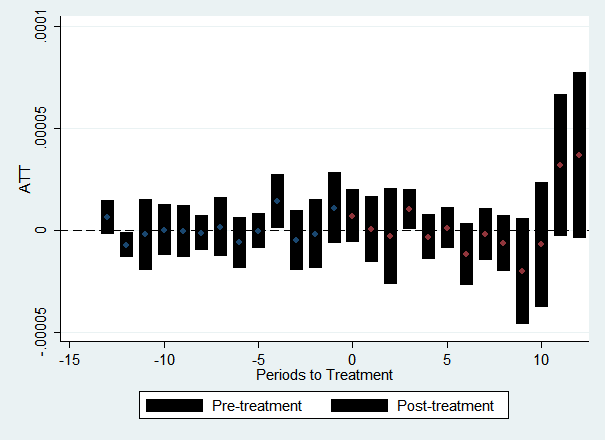


Chart 94 – With covariates: Y7 - Leptospirosis Notifiable diseases - consortia cluster


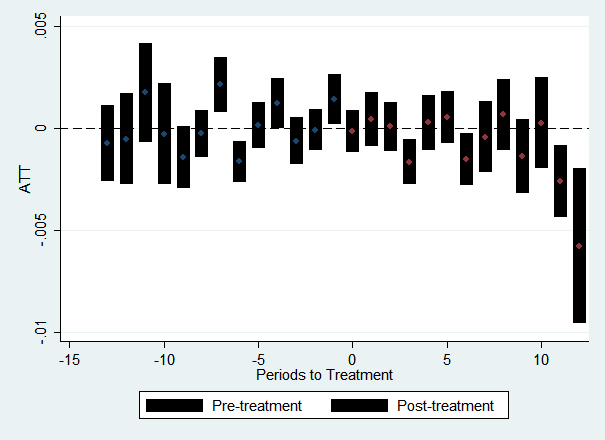


Chart 95 – With covariates: Y8 - Dengue Notifiable diseases - municipal cluster


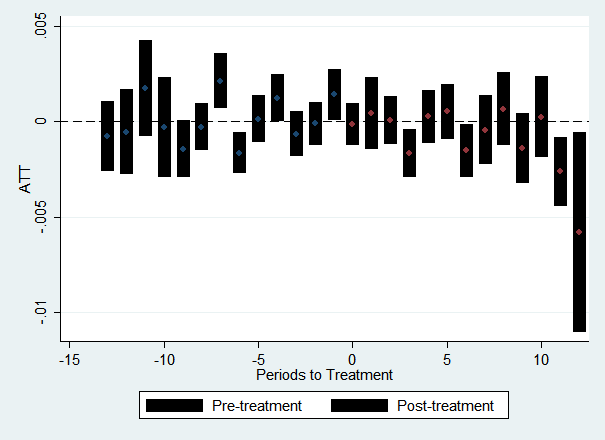


Chart 96 – With covariates: Y8 - Dengue Notifiable diseases - consortia cluster


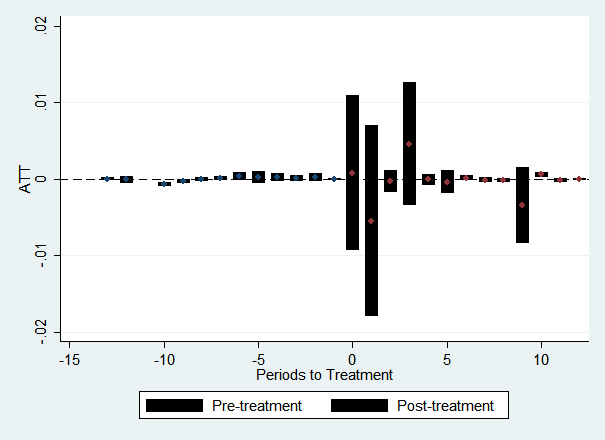


Chart 97 – With covariates: Y9 - Acute Chagas disease Notifiable diseases - municipal cluster


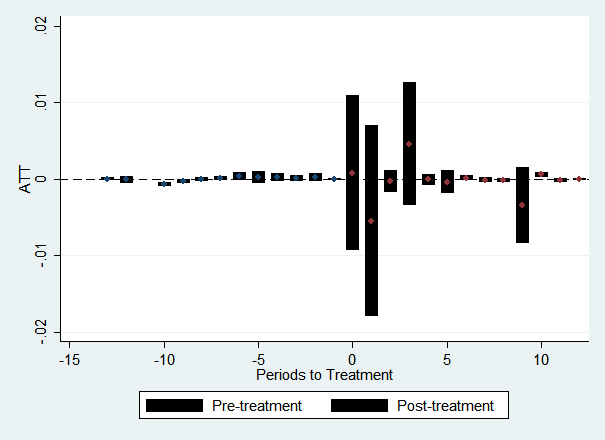


Chart 98 – With covariates: Y9 - Acute Chagas disease Notifiable diseases - consortia cluster


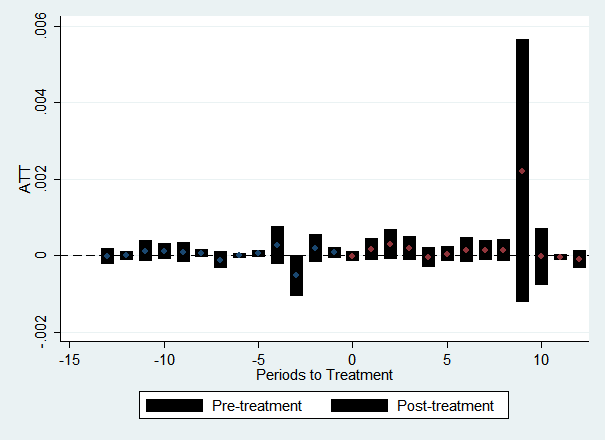


Chart 99 – With covariates: Y10 - Malaria Notifiable diseases - municipal cluster


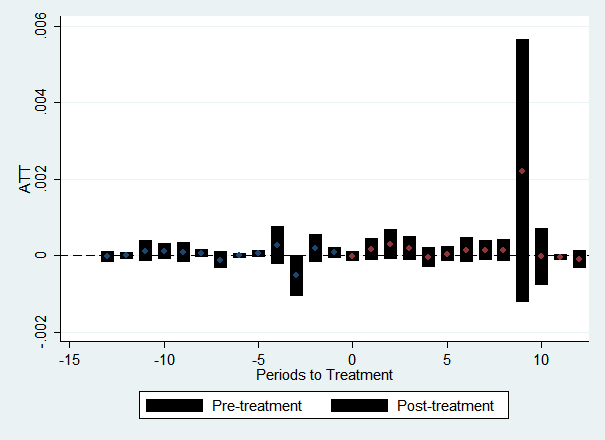


Chart 100 – With covariates: Y10 - Malaria Notifiable diseases - consortia cluster

Chart 101 – With covariates: Y11 - Dermatosis Notifiable diseases - municipal cluster

Chart 102 – With covariates: Y11 - Dermatosis Notifiable diseases - consortia cluster

Chart 103 – With covariates: Y12 - Yellow fever Notifiable diseases - municipal cluster

Chart 104 – With covariates: Y12 - Yellow fever Notifiable diseases - consortia cluster

Chart 105 – With covariates: Y13 - Hantavirus Notifiable diseases - municipal cluster

Chart 106 – With covariates: Y13 - Hantavirus Notifiable diseases - consortia cluster

Chart 107 – With covariates: Y14 - Leishmaniasis Notifiable diseases - municipal cluster

Chart 108 – With covariates: Y14 - Leishmaniasis Notifiable diseases - consortia cluster

Chart 109 – With covariates: Y15 - Whooping Notifiable diseases - municipal cluster

Chart 110 – With covariates: Y15 - Whooping Notifiable diseases - consortia cluster

Chart 111 – With covariates: Y16 - Fetal Growth and Malnutrition - Hospitalizations - municipal cluster

Chart 112 – With covariates: Y16 - Fetal Growth and Malnutrition - Hospitalizations - consortia cluster

Chart 113 – With covariates: Y17 - Diarrhea/gastroenteritis - Hospitalizations(less 5 years) - municipal cluster

Chart 114 – With covariates: Y17 - Diarrhea/gastroenteritis - Hospitalizations(less 5 years) - consortia cluster

Chart 115 – With covariates: Y18 - Diarrhea/gastroenteritis - Hospitalizations(5-9 years) - municipal cluster

Chart 116 – With covariates: Y18 - Diarrhea/gastroenteritis - Hospitalizations(5-9 years) - consortia cluster

Chart 117 – With covariates: Y19 - Diarrhea/gastroenteritis - Hospitalizations(10-14 years) - municipal cluster

Chart 118 – With covariates: Y19 - Diarrhea/gastroenteritis - Hospitalizations(10-14 years) - consortia cluster

Chart 119 – With covariates: Y20 - Diarrhea/gastroenteritis - Hospitalizations(15-19 years) - municipal cluster

Chart 120 – With covariates: Y20 - Diarrhea/gastroenteritis - Hospitalizations(15-19 years) - consortia cluster

Chart 121 – With covariates: Y21 - Diarrhea/gastroenteritis - Hospitalizations(20-29 years) - municipal cluster

Chart 122 – With covariates: Y21 - Diarrhea/gastroenteritis - Hospitalizations(20-29 years) - consortia cluster

Chart 123 – With covariates: Y22 - Diarrhea/gastroenteritis - Hospitalizations(30-39 years) - municipal cluster

Chart 124 – With covariates: Y22 - Diarrhea/gastroenteritis - Hospitalizations(30-39 years) - consortia cluster

Chart 125 – With covariates: Y23 - Diarrhea/gastroenteritis - Hospitalizations(40-49 years) - municipal cluster

Chart 126 – With covariates: Y23 - Diarrhea/gastroenteritis - Hospitalizations(40-49 years) - consortia cluster

Chart 127 – With covariates: Y24 - Diarrhea/gastroenteritis - Hospitalizations(50-59 years) - municipal cluster

Chart 128 – With covariates: Y24 - Diarrhea/gastroenteritis - Hospitalizations(50-59 years) - consortia cluster

Chart 129 – With covariates: Y25 - Diarrhea/gastroenteritis - Hospitalizations(60-69 years) - municipal cluster

Chart 130 – With covariates: Y25 - Diarrhea/gastroenteritis - Hospitalizations(60-69 years) - consortia cluster

Chart 131 – With covariates: Y26 - Diarrhea/gastroenteritis - Hospitalizations(70-79 years) - municipal cluster

Chart 132 – With covariates: Y26 - Diarrhea/gastroenteritis - Hospitalizations(70-79 years) - consortia cluster

Chart 133 – With covariates: Y27 - Diarrhea/gastroenteritis - Hospitalizations(+80 years) - municipal cluster

Chart 134 – With covariates: Y27 - Diarrhea/gastroenteritis - Hospitalizations(+80 years) - consortia cluster

Chart 135 – With covariates: Y28 - Other intestinal diseases - Hospitalizations(less 5 years) - municipal cluster

Chart 136 – With covariates: Y28 - Other intestinal diseases - Hospitalizations(less 5 years) - consortia cluster

Chart 137 – With covariates: Y29 - Other intestinal diseases - Hospitalizations(5-9 years) - municipal cluster

Chart 138 – With covariates: Y29 - Other intestinal diseases - Hospitalizations(5-9 years) - consortia cluster

Chart 139 – With covariates: Y30 - Other intestinal diseases - Hospitalizations(10-14 years) - municipal cluster

Chart 140 – With covariates: Y30 - Other intestinal diseases - Hospitalizations(10-14 years) - consortia cluster

Chart 141 – With covariates: Y31 - Other intestinal diseases - Hospitalizations(15-19 years) - municipal cluster

Chart 142 – With covariates: Y31 - Other intestinal diseases - Hospitalizations(15-19 years) - consortia cluster

Chart 143 – With covariates: Y32 - Other intestinal diseases - Hospitalizations(20-29 years) - municipal cluster

Chart 144 – With covariates: Y32 - Other intestinal diseases - Hospitalizations(20-29 years) - consortia cluster

Chart 145 – With covariates: Y33 - Other intestinal diseases - Hospitalizations(30-39 years) - municipal cluster

Chart 146 – With covariates: Y33 - Other intestinal diseases - Hospitalizations(30-39 years) - consortia cluster

Chart 147 – With covariates: Y34 - Other intestinal diseases - Hospitalizations(40-49 years) - municipal cluster

Chart 148 – With covariates: Y34 - Other intestinal diseases - Hospitalizations(40-49 years) - consortia cluster

Chart 149 – With covariates: Y35 - Other intestinal diseases - Hospitalizations(50-59 years) - municipal cluster

Chart 150 – With covariates: Y35 - Other intestinal diseases - Hospitalizations(50-59 years) - consortia cluster

Chart 151 – With covariates: Y36 - Other intestinal diseases - Hospitalizations(60-69 years) - municipal cluster

Chart 152 – With covariates: Y36 - Other intestinal diseases - Hospitalizations(60-69 years) - consortia cluster

Chart 153 – With covariates: Y37 - Other intestinal diseases - Hospitalizations(70-79 years) - municipal cluster

Chart 154 – With covariates: Y37 - Other intestinal diseases - Hospitalizations(70-79 years) - consortia cluster

Chart 155 – With covariates: Y38 - Other intestinal diseases - Hospitalizations(+80 years) - municipal cluster

Chart 156 – With covariates: Y38 - Other intestinal diseases - Hospitalizations(+80 years) - consortia cluster

Chart 157 – With covariates: Y39 - Expenditures - Sanitation - municipal cluster

Chart 158 – With covariates: Y39 - Expenditures - Sanitation - consortia cluster

Chart 159 – With covariates: Y40 - Expenditures - Environmental - municipal cluster

Chart 160 – With covariates: Y40 - Expenditures - Environmental - consortia cluster
